# A novel *Trichinella spiralis* serine proteinase disrupted gut epithelial barrier and mediated larval invasion through binding to RACK1 and activating MAPK/ERK1/2 pathway

**DOI:** 10.1371/journal.pntd.0011872

**Published:** 2024-01-08

**Authors:** Yan Yan Song, Xin Zhuo Zhang, Bo Ning Wang, Yong Kang Cheng, Xin Guo, Xi Zhang, Shao Rong Long, Ruo Dan Liu, Zhong Quan Wang, Jing Cui

**Affiliations:** Department of Parasitology, Medical College, Zhengzhou University, Zhengzhou, China; University of Liverpool, UNITED KINGDOM

## Abstract

**Background:**

Gut epithelium is the first natural barrier against *Trichinella spiralis* larval invasion, but the mechanism by which larval penetration of gut epithelium is not completely elucidated. Previous studies showed that proteases secreted by *T*. *spiralis* intestinal infective larvae (IIL) degraded tight junctions (TJs) proteins of gut epithelium and mediated larval invasion. A new *T*. *spiralis* serine proteinase (TsSPc) was identified in the IIL surface proteins and ES proteins, rTsSPc bound to the intestinal epithelial cell (IECs) and promoted larval invasion of IECs. The aim of this study was to characterize the interacted proteins of TsSPc and IECs, and to investigate the molecular mechanisms of TsSPc mediating larval invasion of gut mucosa.

**Methodology/Principal finding:**

IIFT results showed natural TsSPc was detected in infected murine intestine at 6, 12 hours post infection (hpi) and 3 dpi. The results of GST pull-down, mass spectrometry (MS) and Co-IP indicated that rTsSPc bound and interacted specifically with receptor for activated protein C kinase 1 (RACK1) in Caco-2 cells. rTsSPc did not directly hydrolyze the TJs proteins. qPCR and Western blot showed that rTsSPc up-regulated RACK1 expression, activated MAPK/ERK1/2 pathway, reduced the expression levels of gut TJs (occludin and claudin-1) and adherent protein E-cad, increased the paracellular permeability and damaged the integrity of intestinal epithelial barrier. Moreover, the RACK1 inhibitor HO and ERK1/2 pathway inhibitor PD98059 abolished the rTsSPc activating ERK1/2 pathway, they also inhibited and abrogated the rTsSPc down-regulating expression of occludin, claudin-1 and E-cad in Caco-2 monolayer and infected murine intestine, impeded larval invasion and improved intestinal epithelial integrity and barrier function, reduced intestinal worm burdens and alleviated intestinal inflammation.

**Conclusions:**

rTsSPc bound to RACK1 receptor in gut epithelium, activated MAPK/ERK1/2 pathway, decreased the expression of gut epithelial TJs proteins and disrupted the epithelial integrity, consequently mediated *T*. *spiralis* larval invasion of gut epithelium. The results are valuable to understand *T*. *spiralis* invasion mechanism, and TsSPc might be regarded as a vaccine target against *T*. *spiralis* invasion and infection.

## Author summary

A novel *T*. *spiralis* serine proteinase (TsSPc) was identified in excretory/secretory proteins (ESP) of intestinal infective larvae (IIL). The purpose of this study was to determine the mechanism of TsSPc action in larval invasion of intestinal epithelium. IIFT showed that natural rTsSPc bound to intestinal epithelial cells (IECs). GST pull-down, co-immunoprecipitation (Co-IP), IIFT and mass spectrometry verified that rTsSPc specifically bound to the RACK1 receptor in Caco-2 cells, activated MAPK/ERK1/2 signaling pathway, as demonstrated by increased expression of RACK1 and phosphorylated ERK1/2 (p-ERK1/2). Binding of rTsSPc to RACK1 and activation of MAPK/ERK1/2 pathway significantly decreased expression of gut epithelium TJs proteins (occludin, claudin-1 and E-cad), paracellular permeability was obviously increased. Besides, HO (RACK1 inhibitor) and PD98059 (ERK1/2 pathway inhibitor) suppressed and abolished the rTsSPc activating ERK1/2 pathway, and abrogated the rTsSPc down-regulating expression of occludin, claudin-1 and E-cad in Caco-2 monolayer and infected murine intestine, impeded larval invasion and improved intestinal epithelial integrity and barrier function, decreased intestinal worm burdens and meliorated intestinal inflammation. The results indicated that TsSPc binding to gut epithelial RACK1 receptor activated MAPK/ERK1/2 pathway, decreased expression of gut TJs proteins, and destroyed gut epithelial integrity, thereby mediated *T*. *spiralis* larval invasion of gut mucosa. rTsSPc might be a candidate vaccine target to block *T*. *spiralis* larval intrusion.

## Introduction

*Trichinella spiralis* is an intestinal and tissue-parasitizing nematode of the genus *Trichinella*, which infects over 150 kinds of mammalian animals in the world [1]. Human *T*. *spiralis* infection is caused by eating raw or semi-cooked animal meat infected with the muscle larvae (ML) [2]. In Argentina and Chile, 6662 and 258 patients with trichinellosis were recorded during 2012–2020 and 2005–2015, respectively [3]. In China, 8 human trichinellosis outbreaks with 479 cases and 2 deaths occurred during 2009–2020, and 7 outbreaks (87.50%) were resulted from infected pork, swine pork is the dominating source of *Trichinella* infection [4]. *T*. *spiralis* infection is not only a significant public health issue, but also an important risk to meat food safety. Therefore, it is necessary to develop anti-*Trichinella* vaccines to block the transmission of *T*. *spiralis* infection among domestic food animals [5].

Once the *T*. *spiralis*-infected meat is ingested by the host, the infected meat is digested by gastric juice in the stomach, and the ML are released from the collage capsule and activated into intestinal infectious larvae (IIL) by the bile and enteral contents. The IIL enter intestinal epithelial cell (IEC) monolayer where they undergo four molts to develop into adult worms (AW). After mating, female adults produce newborn larvae (NBL), which enter the venule and lymphatic vessel and diffuse to the whole body through blood circulation until they reach the final parasited skeletal muscle, and develop into the encapsulated ML to complete the life cycle [6]. The gut mucosal epithelial cells are the first natural defense barrier against the *T*. *spiralis* IIL invasion, and also the priority sites of interaction between intestinal parasites and the host [7]. The IIL successful invasion of the IECs is the most pivotal step for *T*. *spiralis* infecting the host [8,9]. *T*. *spiralis* larvae have no oral appendages or spikes, so the gut epithelial invasion is unlikely due to the larval mechanical penetration; it is likely due to the degradation and destruction of gut epithelium by various proteases in the IIL surface or excretion/secretion proteins (ESP) [10–12].

Intestinal epithelial barrier is crucial for preventing the pathogen invasion and limiting the entry of pathogenic antigens and toxic substances into the systemic circulation. When intestinal barrier is damaged, cell permeability is increased. Some intestinal parasite infection damaged the intestinal barrier, such as *Cryptosporidium*, and *Giardia lamblia* [13]. The cysteine protease secreted by *Giardia* destroyed the tight junctions (TJs) proteins (claudin-1, claudin-4, occludin, JAM-1 and β-Catenin) and adherens junction protein (E-cadherin, E-cad) [14]. A *Giardia*-secreted mature cathepsin B-like enzyme (giardipain-1) degraded the TJs (occludin and claudin-1) of IEC 6 monolayer, and silencing of giardipain-1 gene in *Giardia* trophozoites reduced the damage in cell monolayers [15]. Recombinant *Entamoeba histolytica* cysteine protease affected epithelial adhesion by degrading and delocalizing claudin-1, and claudin-2 expression at TJs [16]. Intestinal helminthes often disrupt the intestinal barrier through mechanical movement, but intestinal nematodes released their ESP to destroy the intestinal barrier. Previous studies showed that *Trichuris suis* ESP reduced intestinal barrier function and inhibited inflammatory cytokine production of IECs [17]. The adult worm ESP from *Haemonchus contortus* and *Teladorsagia circumcincta* disrupted the cultured Caco-2 monolayer and increased the permeability of the cell monolayers, which was neutralized by antibodies from immune host [18]. The exosomes and serine proteases in *T*. *spiralis* ML ESP reduced the expression of the TJs (claudin-1, occludin and ZO-1), increased the epithelial cell monolayer permeability and damaged intestinal barrier [19,20].

The IIL are the first invasive stage in *T*. *spiralis* life cycle, their ESP contain many kinds of proteases. The proteases secreted by the IIL are first exposed to the host’s intestinal mucosa and play an important role in disrupting intestinal epithelial barrier and mediate larval invasion of gut epithelium [21]. By using proteomics/immunoproteomics analysis, various serine proteases were identified in *T*. *spiralis* IIL ESP [7,22]. These serine proteases participated in IIL invasion of host’s gut epithelium [8,23–25]. Therefore, serine proteases may be the important *T*. *spiralis* invasion-related proteases and might be the main candidate target molecules for the vaccines against *T*. *spiralis* invading gut mucosa [26–28].

In previous studies, a new serine proteinase from *T*. *spiralis* (TsSPc, Genbank: U62659.1) was identified in the IIL surface proteins and ESP [10,29]. The serine proteinase was also present in AW and NBL stages of *T*. *spiralis* [30]. The recombinant pQE-80L/TsSPc was constructed, and rTsSPc with his tag was cloned and expressed in our laboratory. We found that rTsSPc specifically bound to the IECs, and promoted larval invasion of IECs [31]. However, rTsSPc bound with what kinds of proteins in IECs was not identified, and the mechanisms of TsSPc promoting larval invasion of IECs are unclear. In this study, to investigate the interaction of TsSPc and IEC proteins, the recombinant expression plasmid pGEX-4T-1/TsSPc was constructed, and rTsSPc with GST tag was prepared. The aim of this study was to characterize the IEC proteins interacting with TsSPc, and to investigate the molecular mechanisms of TsSPc mediating larval invasion of intestinal epithelium at the early stage of *T*. *spiralis* infection.

## Materials and methods

### Ethics statement

This study was conducted according to the National Guidelines for Experimental Animal Welfare (Minister of Science and Technology, People’s Republic of China, 2006). All animal experiments in this study were approved by the Life Science Ethics Committee of Zhengzhou University (No. ZZUIRB GZR 2021–0044).

### *Trichinella* species and experimental animals

A *T*. *spiralis* strain (ISS534) was isolated from a naturally infected pig in Henan Province, China. The *T*. *spiralis* parasite was passaged every 6 months in BALB/c mice with 6–8 weeks old in our laboratory, and each mouse was orally infected with 300 ML [26]. The female BALB/c mice were obtained from the Experimental Animal Center of Zhengzhou University.

### Cell culture

Human colon epithelial cell line Caco-2 was purchased from the Cell Resource Center of Shanghai Institute of Biological Sciences, Chinese Academy of Sciences. Caco-2 cells were cultivated in Eagle medium (MEM)-ALPHA (Sigma-Aldrich, USA), which was supplemented with 10% fetal bovine serum (FBS) (Gibco), 100 U/ml penicillin, 100 μg/ml streptomycin and 100 mM nonessential amino acids (Solarbio, Beijing, China). The cells were inoculated in T25 flasks (NEST, Wuxi, China) and cultured for 7 d. After being digested by trypsin (Solarbio), the cells were again cultured at 37 °C and 5% CO_2_ for 14–21 d to converge on a cover slide in a 6-wells culture plate [14,21].

### Collection of intestinal *T*. *spiralis* worms and ES antigens

The ML was obtained from mouse skeletal muscles infected with *T*. *spiralis* at 42 days post infection (dpi) following artificial digestion [23]. Ten mice were orally infected with *T*. *spiralis* ML (5000 larvae per animal). The infected mice were euthanized at 6 hours post infection (hpi). The upper two thirds of small intestine (duodenum and jejunum) were collected and longitudinally incised, rinsed three times with cold physiological saline, cut into 2-cm-long fragments, and cultivated in normal saline at 37 °C for 2.5 h. Then, the IIL worms released from small intestine to normal saline were collected, and their excretion/secretion (ES) antigens were prepared as described before [11,32]. In brief, after the IIL worms were thoroughly washed with sterile physiological saline and serum-free RPMI 1640 medium (100 U penicillin/ml and 100 μg/ml streptomycin), the worms were cultivated at 37 °C and 5% CO_2_ at 5000 worm/ml medium for 18 h. The culture supernatant was concentrated by using Amicon Ultra-3 centrifugal filtration device (MW cut-off value: 3 kDa), and centrifuged at 4 °C and 5000 × *g* for 3 h. The IIL ES antigens were collected and stored at −80 °C till use [10].

### Expression of rTsSPc and preparation of anti-rTsSPc antibodies

Previous studies reported that rTsSPc with His-tag had good antigenicity. To carry out the subsequent GST pull-down assay, rTsSPc with a GST-tag was also expressed in this study. The full-length cDNA sequence of TsSPc gene (GenBank: U62659.1) was cloned into pGEX-4T-1 to construct recombinant expression plasmid pGEX-4T-1/TsSPc, and recombinant pGEX-4T-1/TsSPc was transformed into *E*. *coli* Origami (Novagen). The expression of rTsSPc was induced with 0.5 mM isopropyl β-D-1-thiogalactopyranoside (IPTG) at 16 °C for 3 d [33], and the rTsSPc was purified with GST-Sefinose Resin 4FF (Settled Resin) (Sangon Biotech., Shanghai, China) [25,34]. The molecular weight (MW) of rTsSPc with GST tag was 51.18 kDa (rTsSPc with 25.18 kDa and GST tag protein with 26 kDa).

Each of fifteen BALB/c mice was immunized subcutaneously with 20 μg rTsSPc emulsified with complete Freund’s adjuvant, then boosted for two times by 20 μg rTsSPc emulsified with incomplete Freund’s adjuvant every 2 weeks [35]. After 2 weeks of final immunization, the tail blood of immunized mice was collected to prepare anti-rTsSPc serum. Anti-rTsSPc antibody IgG was determined using ELISA [36], and anti-rTsSPc IgG titer was 1:10^5^.

### Indirect immunofluorescence test (IIFT)

To assess whether natural TsSPc secreted by *T*. *spiralis* binds to gut epithelium, the IIFT was performed [37]. Briefly, small intestines were collected from mice infected with 300 ML at 6, 12 hpi and 3 dpi, respectively. The infected murine intestine was fixed with 4% paraformaldehyde and embedded in paraffin, and cross-section with a thickness of 2 μm was cut with a microtome [38]. Intestinal sections were blocked using 5% goat serum at 37 °C for 1 h, washed three times with PBS, and then probed with 1:10 diluted anti-rTsSPc serum, infected serum and pre-immune serum at 37 °C for 2 h. Following washes, the sections were incubated with goat anti-mouse IgG-Alexa Fluor 488 conjugate (1:100; Abways, Shanghai, China). After washing again, the sections were examined under a fluorescence microscope (Olympus, Japan) [39,40].

### CCK-8 assay of cell viability

To determine the effect of various dose of rTsSPc on Caco-2 cell viability, CCK-8 assay was conducted as reported before [19]. Caco-2 cells (5 × 10^3^ cells/well) were inoculated in a-96-well plate (100 μl/well). Cells were cultured in MEM media until grown to the confluence [33]. Diverse concentrations of rTsSPc (0, 5, 10, 15, 20, 25 and 30 μg/ml) were added into the plates, and incubated for different times (0, 0.5, 1.0, 1.5, 2.0, 2.5 and 3.0 h). Then, 10 μl CCK-8 solutions were added in each well and cultured for 2 h. The absorbance at 450 nm was measured using a plate reader (Tecan, Switzerland). Cell viability = (OD values of experiment group-OD values of blank control)/(OD values of PBS group-OD values of blank control) × 100%.

### GST pull-down test

GST pull-down test is based on a specific combination of glutathione thiotransferase (GST) and glutathione (GSH) in Sephrose 4B beads. The rTsSPc protein fused with GST tag was purified, and then incubated with whole soluble proteins of Caco-2 cells at 4 °C for 4 h. The conjugates of Caco-2 protein with GST-rTsSPc were captured, and the protein complex was denatured and identified by SDS-PAGE and Western blot [41,42]. Briefly, GST-rTsSPc was first incubated with GSH conjugated resin (Sangon, China). After washes with washing buffer (4.2 mM Na_2_PO_4_, 2 mM KH_2_PO_4_, 140 mM NaCl, 10 mM KCl), the complex was incubated with Caco-2 cell soluble proteins. The only GST tag in the GSH conjugated resin (Sangon) is used as a negative control [43].

### LC-MS/MS, protein identification, annotation and bioinformatic analysis

The two clear Caco-2 cell bands of 36 and 55 kDa captured by GST-pull down were excised from the gel and subjected to in-gel tryptic digestion as previously described [10]. After digestion, Peptide mixtures were separated by high-performance liquid chromatography (HPLC) followed by tandem MS analysis [44]. All MS/MS spectrum data were searched against the *Homo sapiens* protein database of NCBI (https://www.ncbi.nlm.nih.gov/) by using SEQUEST algorithm. Multiple peptide identifications were generally returned by SEQUEST for each MS/MS spectrum and for each parention change state. The protein identification criteria used in this study were based on Delta CN (≥ 0.1) and Xcorr (one charge ≥ 1.9, two charges ≥ 2.2, and three charges ≥ 3.75) [45].

In order to further identify the functions of the proteins screened, their MW and isoelectric points (pI) were calculated using the online compute pI/MW tool (http://web.expasy.org/computepi/). The matched terms were subjected to GO categories using the Web Gene Ontology Annotation Plot (WEGO) (http://wego.genomics.org.cn/cgi-bin/wego/index.pl) [46]. Using WEGO, the annotation items were classified into cellular components, molecular functions, and biological processes according to GO hierarchy. And then, quantitative statistics were conducted on the MW and pI of the corresponding proteins [11].

### Co-localization of rTsSPc and RACK1 in Caco-2 cells by IIFT

To investigate co-localization of rTsSPc and receptor for activated protein C kinase 1 (RACK1) in Caco-2 cells, the IIFT was performed as reported before [47,48]. Briefly, confluent Caco-2 monolayers were apically incubated with 20 μg/ml of rTsSPc for 2 h at 37 °C. IIL ES antigens were used as a positive control and PBS as a negative control. After interaction, the cells were washed three times with washing buffer (140 mM NaCl, 2.7 mM KCl, 10 mM Na_2_HPO_4_, 1.8 mM KH_2_PO_4_, pH 6.8) to eliminate unbound protein molecules. The cells were fixed in 4% paraformaldehyde at room temperature for 15 min. To reduce non specificity, cells were blocked for 2 h with 1% bovine serum albumin (BSA; Sigma). Then, the cells were probed overnight at 4 °C by rabbit anti-human RACK1 antibody (1:1 000; Servicebio, Wuhan, China) and mouse anti-rTsSPc immune serum (1:100). Then, the cells were stained by using Alexa Fluor 488 labeled goat anti-mouse IgG (1:100; Abways, Shanghai, China) and CY3 labeled goat anti-rabbit IgG (1:100; Servicebio) as the secondary antibody. After being washed with PBST, a 4′,6-diamidino-2-phenylindole (DAPI) was used for fluorescence staining of the cell nucleus. Finally, the cells were observed under a laser confocal fluorescence microscopy [24,42,49].

### Co-immunoprecipitation (Co-IP)

Co-IP was based on specific binding of the protein A/G agar-agar system and IgG antibodies. The results of GST pull-down analysis were mutually validated with Co-IP test. To verify whether rTsSPc is able to bind to RACK1 in Caco-2 cells, Co-IP was performed as previously reported [50–52]. In brief, 20 μg/ml rTsSPc was first incubated with Caco-2 cells at 37 °C for 2 h, and soluble cellular proteins were prepared with DISC lysis buffer (30 mM pH8.0 Tris-HCl, 120 mM NaCl, 10% glycerol, 1% TritonX-100). After centrifugation at 12,000 *g* for 5 min, protein A/G pre-coupled with anti-mouse IgG antibody was added to the protein supernatant of Caco-2 cells, and incubated at 4 °C for 30 min to remove the nonspecific binding proteins, then centrifuged at 1 000 *g* for 1 min, protein A/G pre-coupled with anti-rTsSPc antibodies was added to Caco-2 cell protein supernatant and incubated at 4 °C for 6 h. Caco-2 cell protein incubated with normal mouse IgG coupled with protein A/G was used as a negative control. The beads were washed three times with DISC lysis buffer, and the bound protein complex (protein A/G-rTsSPc-RACK1) was eluted from the beads and denatured by boiling for 5 min. The eluted protein complex was subjected to 12% SDS-PAGE and transferred to PVDF membrane (Millipore, USA). The membrane was blocked at 37 °C for 2 h using 5% skimmed milk in Tris-buffered saline containing 0.05% Tween (TBST), and cut into the strips. The strips were probed at 4 °C overnight with anti-RACK1 antibody (1:1 000 dilutions; Servicebio) and anti-rTsSPc serum (1:100 dilutions). After washing with TBST, the strips were incubated with HRP conjugated goat anti-rabbit IgG or HRP-conjugated goat anti-mouse IgG (1:10 000; Southern Biotech, USA) incubated at 37 °C for 1 h. After washes again, the color was developed using an enhanced chemiluminescent kit (AEC, Solarbio, Beijing, China) [53,54].

### IIFT analysis of gut epithelium TJs disruption caused by rTsSPc

To ascertain whether rTsSPc regulates the TJs protein expression of gut epithelium, Caco-2 cells were cultured on a cover glass slide until grown to the confluence [21,24]. The cell monolayer was incubated with 20 μg/ml rTsSPc at 37 °C for 2 h, simultaneously, rTsSPc+PMSF (serine protease inhibitor) and heating inactivated rTsSPc were used as negative controls, trypsin and IIL ES antigens (containing serine protease and other proteases) were used as the positive controls [21]. After being washed with PBS, the monolayers were fixed with 4% formaldehyde for 20 min. After washes, the monolayers were permeabilized for 10 min with 0.25% Triton X-100 saponin in PBS and blocked with 1% BSA (Sigma). The monolayers were probed overnight at 4 °C with rabbit anti-human polyclonal antibodies against occludin (1:160), claudin-1 (1:16) or claudin-2 (1:125) (Thermo Fisher, USA) or a rat anti-human monoclonal antibody against E-cad (1:100; Santa Cruz, USA), and then incubated with Cy3-conjugated goat anti-rabbit IgG (1:100) or FITC-conjugated goat anti-rat IgG (1:100; Servicebio) at 37 °C for 1 h. Finally, the monolayers were incubated for 5 min with DAPI to specifically stain cell nuclei [16,55]. Upon completion of the staining procedure, the coverslips were transferred to glass slides and observed on fluorescence microscopy. Moreover, the intercellular localization of TJs proteins was further examined under a fluorescence microscopy and analyzed with Olympus Fluoview software. Finally, the intercellular content of TJs proteins in Caco-2 monolayer was semi-quantitatively analyzed using ImageJ software for analysis [56].

### Real-time quantitative PCR (qPCR) assay

To investigate that rTsSPc regulates the transcription levels of TJs proteins in Caco-2 cells, qPCR was conducted as previously reported [22]. Caco-2 monolayer was co-incubated with rTsSPc (20 μg/ml) at 37 °C for 2 h, and total RNAs were extracted from treated Caco-2 cells using TRIzol (Invitrogen, USA). The RNAs were reverse-transcribed into cDNA using Hifair V one-step RT-cDNA digestion SuperMix for qPCR kit (YESEN, Shanghai, China) as templates for qPCR. qPCR was performed by using Hieff UNICON Universal Blue qPCR SYBR Green Master Mix (YESEN) to amplify the cDNA. Transcription levels of E-cad, occludin, claudin-1 and claudin-2 in Caco-2 cells and infected murine intestine were ascertained by qPCR, and transcription levels of intestinal pro-inflammatory cytokines (TNF-α and IL-1β) and anti-inflammatory cytokines (IL-4 and IL-10) in infected murine intestine were assessed by qPCR. The specific primers for qPCR were shown in S1 Table. The relative transcription level of the related genes was normalized by subtracting the transcription of a housekeeping gene GAPDH. Transcription level of the target genes was calculated with the 2^−ΔΔCt^ method as previously reported [42,44,57].

### Western blot analysis

To further identify the interaction between rTsSPc and RACK1, Western blotting was performed as previously reported [5,23]. Caco-2 cells were incubated with rTsSPc for 2 h at 37 °C, rTsSPc+PMSF, heating inactivated rTsSPc, trypsin, and IIL ES antigens were set as controls. After incubation, Caco-2 cell proteins were subjected to SDS-PAGE and transferred to PVDF membrane (Millipore). Subsequently, the membranes were sealed with 5% skimmed milk. After washes with TBST, the membranes were probed by rabbit anti-human RACK1 antibody (1: 1 000) and rabbit anti-human GAPDH antibody (1: 1 000; Servicebio). Alternatively, the membrane was probed overnight at 4 ° C with rabbit anti-human p-ERK1/2 (1: 1 000; Abmart, Shanghai, China) or rabbit anti-human ERK1/2 antibody (1:1 000, Abmart). Following washes again, HRP-labeled goat anti-rabbit or anti-mouse IgG conjugates were used as secondary antibodies. The membrane was colored by an ECL enhanced chemiluminescence kit (CWBIO, Beijing, China), and Image J was used for protein band analysis [58]. Moreover, to assess the hydrolysis or regulation role of rTsSPc on TJs proteins of Caco-2 monolayer, the rTsSPc pre-incubated cell monolayer was also probed by rabbit antibodies against occludin (1:500), claudin-1 (1:200), claudin-2 (1:200) or E-cad (1:100) (ThermoFisher, USA) overnight at 4 °C, and an anti-GAPDH antibody (1:1000) was used as an internal control [34,59].

### Western blotting of direct hydrolysis of TJs in Caco-2 cell proteins by rTsSPc

To further confirm whether rTsSPc disrupting gut epithelial integrity is resulted from reducing expression of TJs proteins in Caco-2 cells, or from the rTsSPc’s direct hydrolysis on TJs proteins, the *in vitro* rTsSPc degradation on TJs proteins was performed as previously reported [21]. Soluble Caco-2 cell proteins (20 μg) were incubated with 2 μg rTsSPc at pH 8.0 and 37 °C for 16 h; 1 μg trypsin and 15 μg IIL ES antigens were served as positive controls, while recombinant enzymatic active site mutation TsSPc (MTsSPc), inactivated rTsSPc and PBS were set as negative controls. Subsequently, the quantity of TJs proteins in Caco-2 cell proteins was measured by Western blot [59].

### Assay of transepithelial electrical resistance (TEER) and paracellular permeability

The integrity of Caco-2 monolayer after cells became fully confluence was first assessed through measuring TEER with an EVOM epithelial voltmeter (Millipore, USA) [16,19,55]. Briefly, Caco-2 cells (1 × 10^5^/well) were cultivated at 37 °C and 5% CO_2_ in a 24 well Trans-well plate with insert (pore size of 0.4 μm) for 21 d. Each well was measured three times and calculated the mean resistance value of each well. Moreover, each test included a cell-free blank well. TEER (%) = (test group value-blank group value)/(PBS group value-blank group value) × 100%. The Caco-2 monolayers with epithelial resistance above 500 Ω•cm^2^ were used for following experiments. Three repetitions were performed for each group of each experiment. To evaluate the rTsSPc damage to the integrity of Caco-2 monolayer barrier, FITC-dextran of 4 kDa (Sigma) was used in this study. The cells were apically treated with rTsSPc (0, 5, 10, 15, 20 and 25 μg/ml) at 37 °C for 2 h. Then the culture medium containing dextran (0.5 mg/ml) was added to the upper chamber, PBS was added to the lower chamber, the solution in lower chamber was collected at 0, 30, 60, 90 and 120 min after cultivation [16]. The sample from the lower chamber was added to a black 96 well plate, and the emission values of the sample were measured with excitation and emission wavelengths of 492 nm and 520 nm. The emission value of dextran through Caco-2 cell monolayer was converted into concentration through a standard curve and compared to the PBS group [19,60].

### Determination of ERK1/2 MAPK pathway in Caco-2 cells by using inhibitor and antibody

To confirm that rTsSPc binding to RACK1 damaged the TJs integrity through activating ERK1/2 MAPK pathway and down-regulating expression of TJs proteins, RACK1 inhibitor harringtonolide (HO; 40 μM; MedChemExpress, USA) and ERK1/2 pathway inhibitor PD98059 (20 μM; MCE, USA) were used in this study [61,62]. Caco-2 cells were pre-treated with the two inhibitors at 37 °C for 24 h, respectively. Additionally, rabbit anti-human RACK1 antibody (1: 100; Servicebio) was also used to block the RACK1 receptor of Caco-2 cells. Then, the inhibitors-treated or antibody-blocked cells were incubated with rTsSPc (20 μg/ml) at 37 °C for 2 h. The IIFT was performed to investigate the expression and location of TJs proteins in rTsSPc-incubated Caco-2 cells [33]. Total RNAs were extracted from treated Caco-2 cells using TRIzol (Invitrogen), and expression levels of TJs mRNA in treated Caco-2 cells were ascertained by qPCR. Moreover, after the above-treated Caco-2 cells were washed using TBST, soluble cell proteins were prepared and Western blot were carried out, and rabbit antibody against occludin, claudin-1, claudin-2 and rat anti-E-cad antibody were used to detect the expression of TJs proteins in rTsSPc-incubated Caco-2 cells which were pre-treated with inhibitors or pre-blocked with antibody [21].

### The *in vitro* larval invasion assay

To determine the role of TsSPc in *T*. *spiralis* IIL invasion of intestinal epithelium, an *in vitro* invasion assay was conducted as described earlier [63,64]. In brief, the MLs were activated to the IILs with 5% porcine bile at 37 °C for 2 h. Caco-2 monolayer was pre-incubated with anti-RACK1 antibody, HO, PD98059 or HO+PD98059 at 37 °C for 24 h. One hundred IILs were added to semi-solid culture medium (serum-free DMEM with 1.75% agarose). After being incubated at 37 °C and 5% CO_2_ for 2 h, the larval invasion of cell monolayer was examined under a light microscopy. The invaded larvae exhibited the snake-like movement and migrated in the monolayer, whereas non-invaded larvae showed spirally coiled on the surface of the cell monolayer [25,65].

### Animal challenge experiment

One hundred and twenty-five mice were randomly divided into 5 groups (25 mice each group). Each group of mice was intraperitoneally injected with 100 μl of the following inhibitor and control: PBS, 50 μl PEG300 (the solvent of HO and PD98059) + 50 μl PBS, 0.5 μg RACK1 antagonist HO per gram of body weight, 10 μg ERK1/2 pathway inhibitor PD98059 per gram of body weight, and 0.5 μg HO+10 μg PD98059 [61]. All mice received three doses of inhibitors (once every other day). At 12 h after injection, all mice were orally challenged with 300 *T*. *spiralis* ML. Ten mice of each group were euthanized at 12 hpi, and intestinal IIL was collected and numbered from infected mice as described previously [22]. The remaining 15 mice in each group were killed at 5 dpi, and intestinal adults were collected from 10 infected mice. The reduction of adult burden was evaluated based on the average number of intestinal adults in the inhibitor group compared to the PBS group [48].

At 5 dpi, the intestine of additional 5 infected mice were collected, and the expression levels of TJs proteins (E-cad, occludin, claudin-1, and claudin-2) and inflammatory cytokines (TNF-α, IL-1β, IL-4 and IL-10) in infected mouse intestines were ascertained by qPCR. Soluble proteins were prepared from intestinal tissue of infected mice, and the expression levels of RACK1 and TJs proteins in intestinal epithelium were ascertained by Western blot [53].

### Intestinal permeability assay in infected mice

The changes in intestinal TJs proteins directly affect intestinal permeability, and 4 kDa FD-4 is commonly used to measure the gut permeability *in vivo*. To evaluate intestinal permeability change caused by *T*. *spiralis* infection, intestinal permeability assay was performed [66]. Briefly, the amount of 4 kDa FITC glucan (FD 4) was measured in the plasma of infected mice. Six mice of each group were pre-treated with HO, PD98059, or HO+PD98059, and then challenged with 300 *T*. *spiralis* ML, and normal mice were served as negative control. At 5 dpi, all mice were fasted and were deprived from water overnight, and each mouse was administered by gavage with 100 μl FD 4 with a dose of 5 mg per mouse. Then the mice started drinking water again. Four hours later, mouse blood was collected and blood plasma was isolated in the dark. The plasma was diluted with PBS in a 1:1 ratio, and the absorbance at 490 nm excitation wavelength and 520 nm emission wavelengths were measured by a microplate reader (Tecan, Switzerland) [20].

### Statistical analysis

All data were analyzed using SPSS 22.0 software. The results are displayed as mean ± standard deviation (SD). One-way ANOVA was used to analyze the difference of transcription and expression levels of TJs proteins, RACK1, p-ERK1/2, inflammatory cytokines and intestinal permeability among different groups. *P* < 0.05 is considered statistically significant.

## Results

### Western blot identification of rTsSPc

Following induction with IPTG, the fusion protein rTsSPc with GST-tag was expressed in *E*. *coli* Origami harboring pGEX-4T-1/TsSPc. The rTsSPc was purified using a GST-Sefinose Resin 4FF. SDS–PAGE analysis showed that rTsSPc had a clearly single band, and its MW was consistent with its predicted size (51.18 kDa: rTsSPc with 25.18 kDa and GST-tag with 26 kDa). Western blot results revealed that rTsSPc with GST-tag was recognized by anti-rTsSPc serum and *T*. *spiralis*-infected murine serum, suggesting that rTsSPc with GST-tag also had good antigenicity (S1 Fig).

### Binding of native TsSPc with gut epithelium

**Fig 1:**
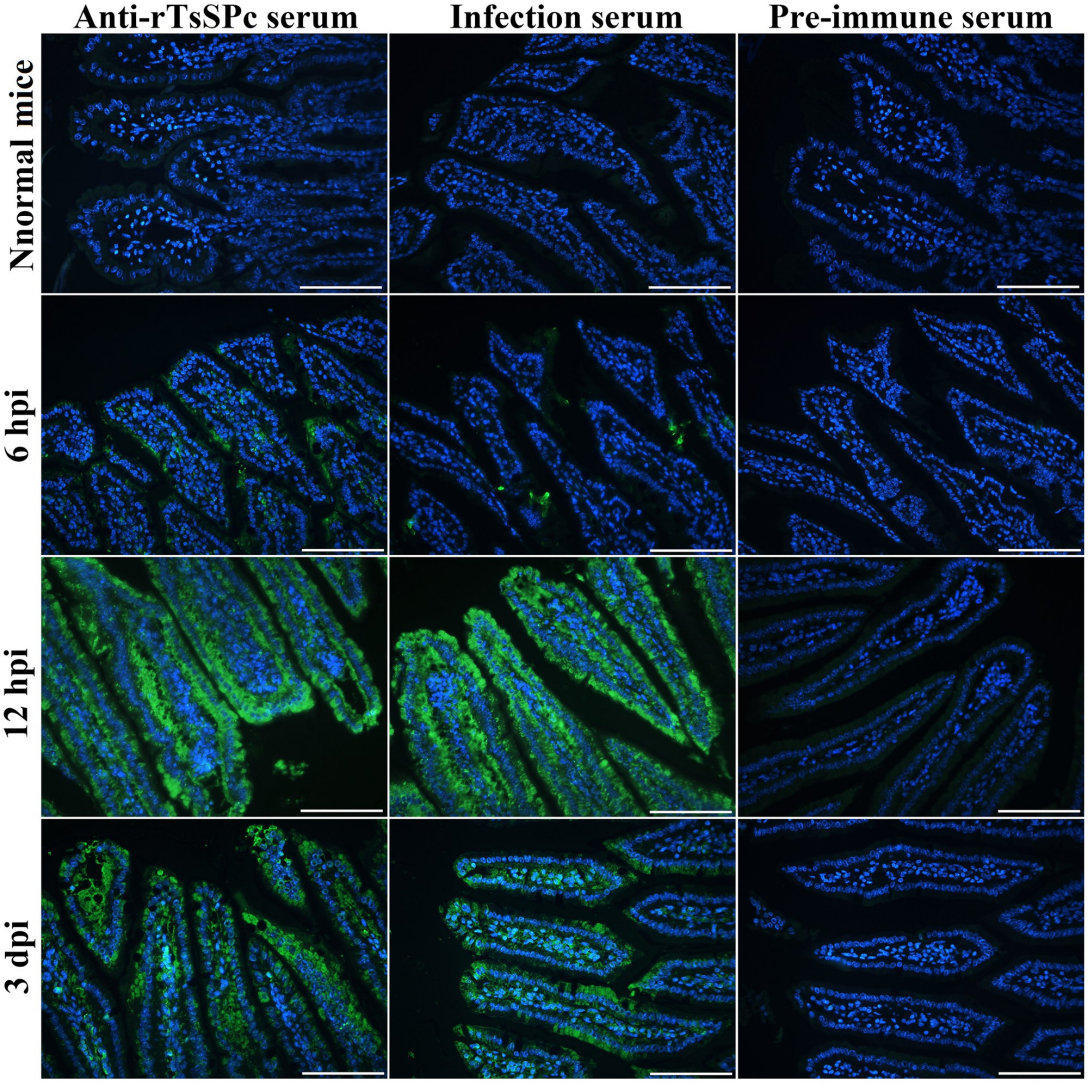
Binding of native TsSPc with enteral epithelium of *T*. *spiralis*-infected mice at various times after infection. Intestinal sections from infected mice at different times after infection were probed by anti-rTsSPc immune serum and infection serum, and green fluorescence immunostaining on enteral epithelium was detected at 6 and 12 hpi, and 3 dpi. Cell nuclei were stained blue with DAPI. Each experiment was performed in triplicate. Scale bars: 100 μm.

The IIFT results showed that when intestinal cross sections from infected mice at different times after infection were probed with anti-rTsSPc serum, green fluorescence immunostaining was observed on gut epithelium at 6 hpi, more strong fluorescence was detected at 12 hpi and 3 dpi (Fig 1), suggesting that natural TsSPc was secreted and bound to the gut epithelium at early intestinal stage of *T*. *spiralis* infection, and TsSPc might participate in larval invasion of host’s gut mucosa.

### The effect of rTsSPc on Caco-2 cell viability

The results of CCK-8 assay showed that 5–25μg/ml of rTsSPc and incubation for 2 h had no obvious effect on Caco-2 cell viability. Only 30 μg/ml of rTsSPc reduced the cell viability compared to the PBS control group (*F* = 5.189, *P* < 0.01). When Caco-2 cells were treated with 20 μg/ml of rTsSPc for 0.5–3.0 h, no evident changes of cell viability were observed (*F* = 0.1321, *P >* 0.05) (S2 Fig). Therefore, Caco-2 cells incubated with 20 μg/ml of rTsSPc for 2 h were used in the following experiment.

### Interaction between rTsSPc and Caco-2 cell proteins assayed by GST pull-down

**Fig 2:**
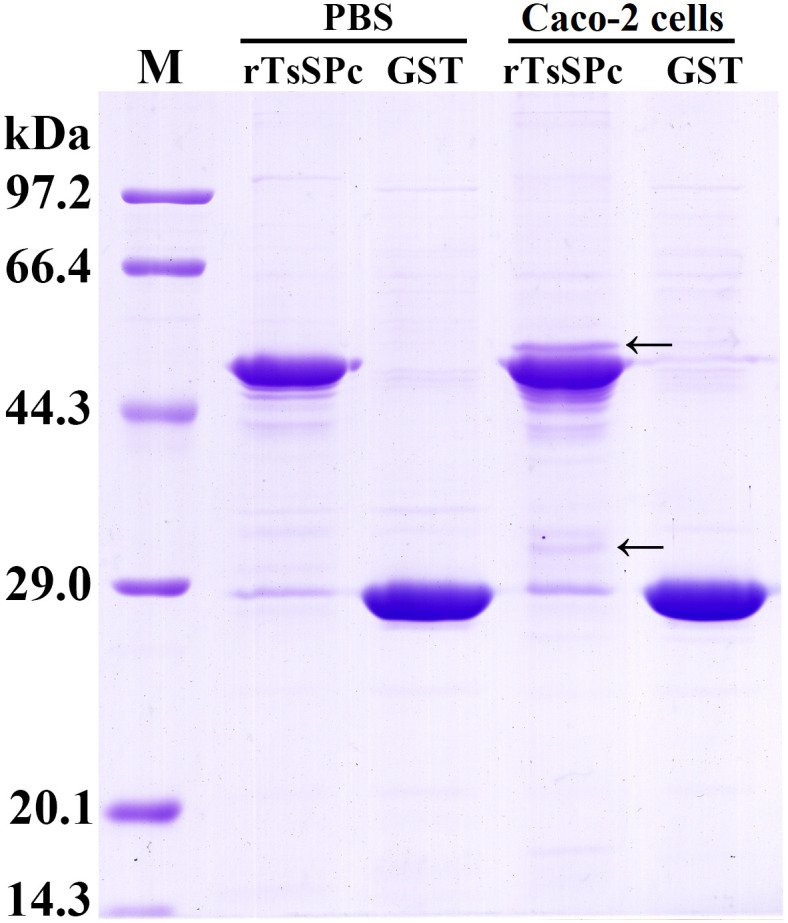
Binding proteins of rTsSPc and Caco-2 cells by GST-pull down. rTsSPc was co-incubated with soluble Caco-2 cell proteins to capture their interacting proteins. M: Low molecular weight protein marker. Each experiment was performed in triplicate. The arrow points to two protein bands with 36 and 55 kDa from the Caco-2 cell proteins captured and interacted with rTsSPc.

The results of GST pull-down assay showed that two additional clear bands of 36 and 55 kDa were observed in the co-incubated lanes of rTsSPc and Caco-2 (Fig 2). The two bands were not observed in the co-incubated lanes of GST and rTsSPc, and GST and Caco-2 cell proteins. The results suggested that the two protein bands captured by GST pull-down were specific binding proteins of rTsSPc and Caco-2 cell proteins.

### Mass spectrometry, GO annotation and theoretical two-dimensional distribution of identified proteins

The possible interacted proteins between rTsSPc and Caco-2 cells were listed in S2 Table. They were RACK1, Protein POF1B (POF1B), Desmocolin 1 (DSC1), UPF0764 protein C16orf89 (146556), Plakophilin 1 (PKP1), Chloride intracellular channel protein (CLIC), and Prohibitin (PHB2).

To further ascertain the functions of the proteins identified, annotation terms were classified into cellular component, molecular function and biological process according to GO hierarchy using WEGO. The MW and pI analysis of specific proteins showed that total of 105 proteins from Caco-2 cells were identified, and the proteins with 10–60 kDa accounted for 71.80%, and proteins with pI 5–10 accounted for 84.76% of the total proteins (S3 Fig). The coverage rate of RACK1 was as high as 22.4%, which was the highest in MS-identified Caco-2 cell proteins, suggesting that rTsSPc likely interacted with RACK1 and participated in regulating the expression of TJs protein in intestinal epithelium. Therefore, RACK1 was selected for the Co-IP assay and subsequent experiments.

### IIFT and Co-IP detection of co-localization and interaction of rTsSPc and RACK1 in Caco-2 cells

**Fig 3:**
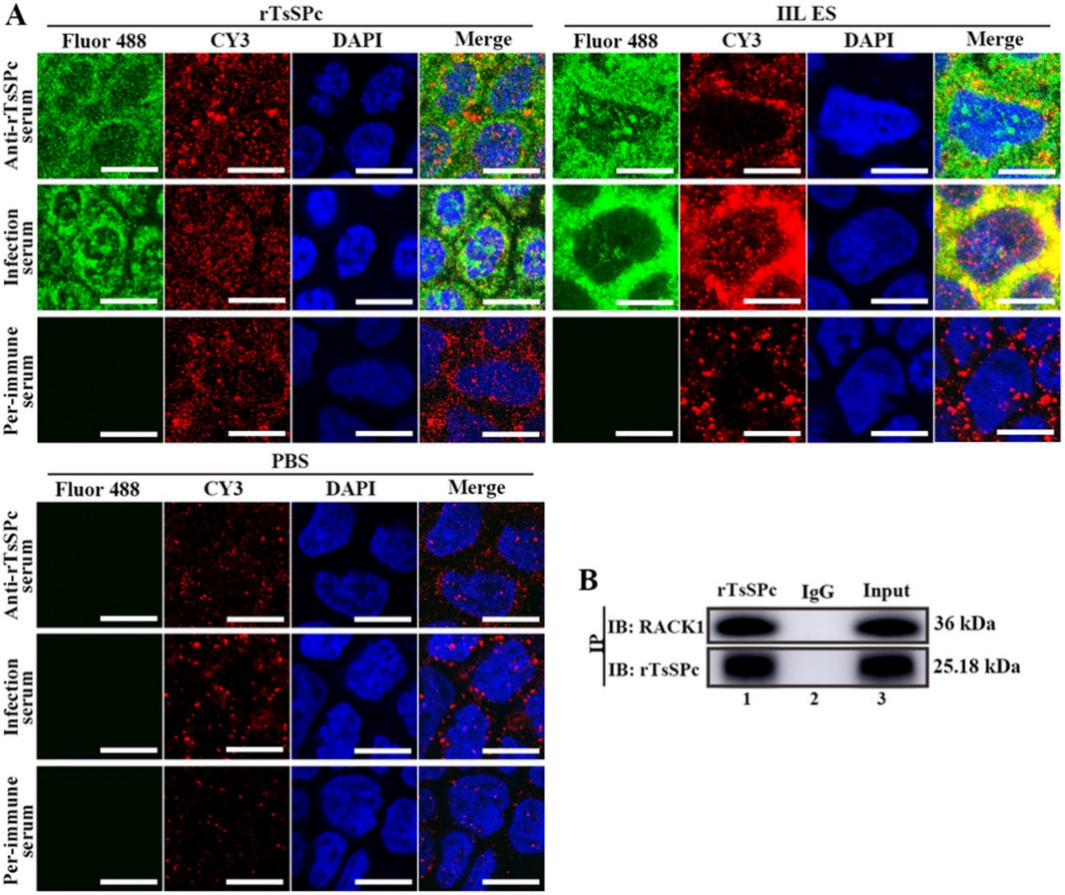
IIFT and Co-IP detection of co-localization and interaction of rTsSPc and RACK1 in Caco-2 cells. **A**: IIFT assay of co-localization of rTsSPc and RACK1 in Caco-2 cells. Caco-2 cells were pre-incubated with rTsSPc and IIL ES antigens at 37 °C for 2 h. After washing, the Caco-2 cells was probed by anti-RACK1 antibody, anti-rTsSPc serum, infected serum, or pre-immune serum, and then stained with goat anti-mouse IgG-Alexa Fluor 488 conjugate and goat anti-rabbit IgG-CY3 conjugate. The cellular nuclei were stained as blue with DAPI. Scale bars: 10 μm. **B**: Co-IP assay of interaction between rTsSPc and RACK1 in Caco-2 cells. Caco-2 cells were first incubated with rTsSPc. Then, anti-rTsSPc antibodies and protein A/G were added and incubated for 6 h. The bound proteins were separated on SDS-PAGE and transferred to the PVDF membrane. The membrane was probed by anti-rTsSPc antibody and anti-RACK1 antibody. Lane 1: Immune co-precipitation complex (rTsSPc, anti-rTsSPc antibody and RACK1 in Caco-2 cell protein); Lane 2: Normal mouse IgG; Lane 3: Caco-2 cell protein after incubation with rTsSPc. Each experiment was carried out in triplicate.

After Caco-2 cells were incubated with rTsSPc, co-localization of rTsSPc and RACK1 in Caco-2 cells was investigated by IIFT. The natural RACK1 in Caco-2 cells was stained red by Cy3, rTsSPc bound to Caco-2 cells showed green fluorescence, and the cell nucleus showed blue fluorescence. The results showed that in rTsSPc-incubated Caco-2 cells, both rTsSPc and RACK1 was co-localized at cell–cell junctions as orange (Fig 3A). The results suggested that rTsSPc had a specific interaction with the RACK1 receptor in Caco-2 cells, and it was also found that RACK1 red fluorescence was strengthened in Caco-2 cells treated with rTsSPc, suggesting that rTsSPc binding to RACK1 in Caco-2 cells activated and up-regulated expression levels of RACK1.

When anti-rTsSPc antibodies and anti-RACK1 IgG were used to probe the lysate of Caco-2 cells incubated with rTsSPc, co-precipitates of rTsSPc and RACK1 in Caco-2 cells were detected by anti-rTsSPc antibodies and anti-RACK1 antibody, respectively; but the two proteins were not detected by normal mouse IgG group (Fig 3B). The Co-IP results indicated that rTsSPc bound specifically to RACK1, and the binding might activate the downstream signaling pathways.

### Gut epithelial TJs protein disruption caused by rTsSPc

**Fig 4:**
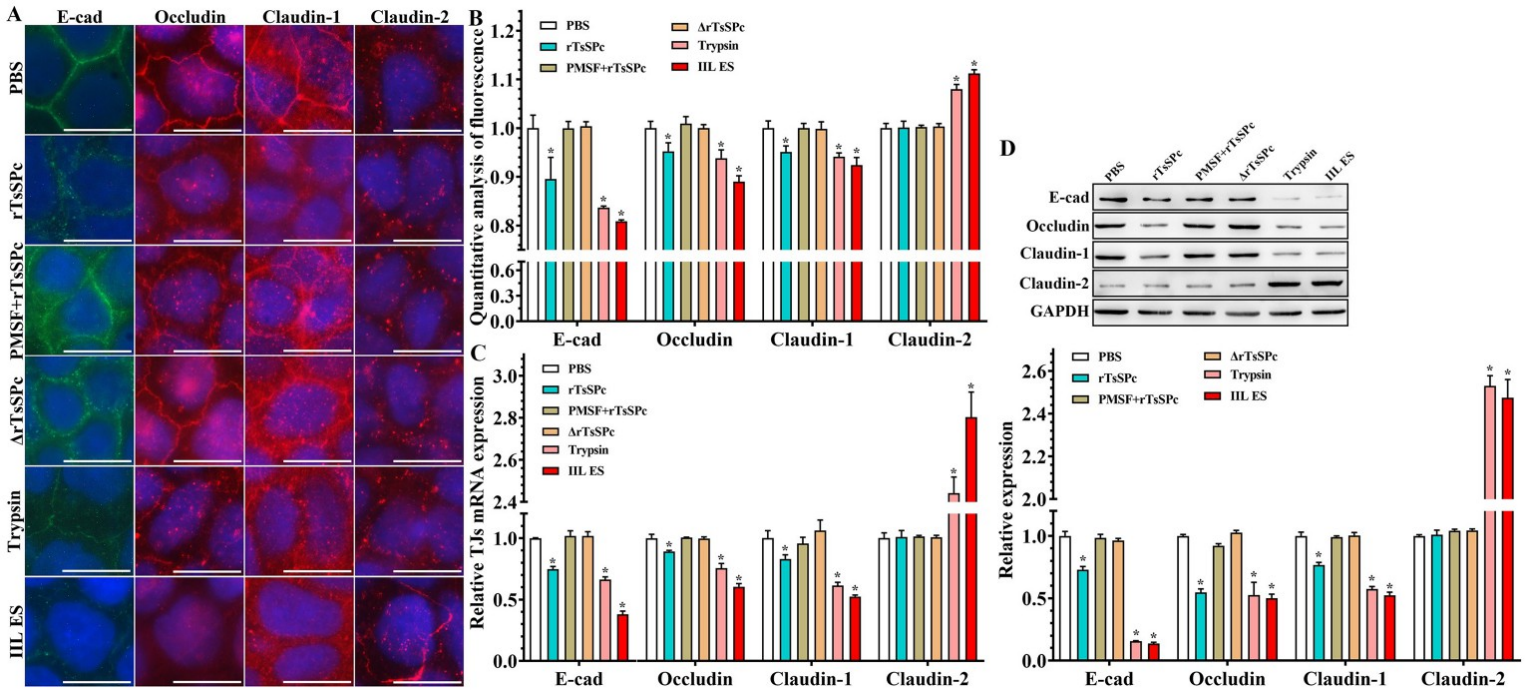
Expression levels of TJs protein in Caco-2 cell monolayers incubated with rTsSPc by IIFT and qPCR and Western blot. **A**: Caco-2 cell monolayers were incubated at 37 °C for 2 h with rTsSPc (20 μg/ml). ΔrTsSPc represents the heating inactivated rTsSPc. PMSF+rTsSPc represents rTsSPc after treated with PMSF (serine protease inhibitor). Trypsin and IIL ES antigens were used as the positive control to evaluate the hydrolyzing or regulatory effect of rTsSPc on tight junctions (TJs) expression in Caco-2 cells. The cells were fixed with 4% paraformaldehyde, permeabilized for 10 min with 0.25% Triton X-100 and blocked with 1% BSA. After washing, the cells were probed with primary antibodies to occludin, claudin-1, claudin-2 or E-cad and then incubated with FITC- or Cy3-conjugated secondary antibodies. Cell nuclei were stained blue with DAPI. The cells were observed under a fluorescence microscope. Scale bars: 10 μm. **B**: Quantitative analysis of the fluorescence. **C**: qPCR analysis of transcription level of TJs protein in rTsSPc-treated Caco-2 cells. **D**: Western blot analysis of expression levels of TJs proteins in rTsSPc-treated Caco-2 cells. Density metrology analysis of protein expression of E-cad, occludin, claudin-1 and claudin-2 obtained from Western blotting compared to the GAPDH expression. Each experiment was carried out in triplicate. * in the Figures represents *P* < 0.0001 compared to the PBS group.

The IIFT results revealed that occludin, claudin-1 and E-cad were distributed at cell–cell junctions in the PBS group; there was a small amount of claudin-2 around the cells (Fig 4A). After treatment with rTsSPc, the amount of occludin, claudin-1 and E-cad around the cells was significantly decreased, and the continuous staining at the cell edge was disappeared. Furthermore, semi-quantitative fluorescence analysis showed that compared to the PBS group, the occludin content in the rTsSPc, trypsin group and IIL ES antigen group was decreased by 0.048, 0.062 and 0.110 folds, respectively (*F* = 32.791, *P* < 0.0001); claudin-1 in the three groups was decreased by 0.049, 0.059, and 0.076 folds, respectively (*F* = 22.191, *P* < 0.0001); E-cad in the three groups was decreased by 0.158, 0.182, and 0.214 folds, respectively (*F* = 48.927, *P* < 0.0001). However, claudin-2 content was respectively increased by 0.080 and 0.113 folds only in trypsin and IIL ES antigen groups (*F* = 96.046, *P* < 0.0001). But, the contents of the four TJ proteins in the two groups of rTsSPc+PMSF and heating inactivated rTsSPc had no statistical difference compared to the PBS group (*P* > 0.05) (Fig 4B). The results suggested that active rTsSPc accelerated the delocalization, degradation or down-regulation of occludin, claudin-1 and E-cad in the cells, but expression of claudin-2 around Caco-2 cells was not obviously changed after treatment with rTsSPc.

The qPCR results showed that after Caco-2 cells were treated with rTsSPc, compared to the PBS group, transcription level of E-cad, occludin, and claudin-1 was decreased by 0.25, 0.11 and 0.17 folds, respectively (*F*_E-cad_ = 263.29, *F*_occludin_ = 145.782, *F*_claudin-1_ = 56.474, *P* < 0.0001), while the transcription level of claudin-2 was not statistically changed (*F* = 511.327, *P* > 0.05) (Fig 4C). The qPCR results indicated that rTsSPc significantly decreased transcription levels of the TJs (occludin, claudin-1, and E-cad), but did not impact evidently on transcription levels of claudin-2.

Western blot analysis exhibited the expression levels of TJs proteins in Caco-2 monolayers treated with rTsSPc (Fig 4D). Compared to the PBS group, rTsSPc obviously hydrolyzed or down-regulated the expression of E-cad, occludin and claudin-1 (*F*_E-cad_ = 1008.1978, *F*_occludin_ = 89.096, *F*_claudin-1_ = 304.598, *P* < 0.0001), but rTsSPc had no evident effect on expression levels of claudin-2 (*P* > 0.05). The results suggested that rTsSPc hydrolyzed or down-regulated the expression of E-cad, occludin and claudin-1, disrupted the integrity of gut epithelial barrier, and consequently mediated the *T*. *spiralis* IIL invasion of host gut mucosa.

### rTsSPc up-regulated RACK1 expression

**Fig 5:**
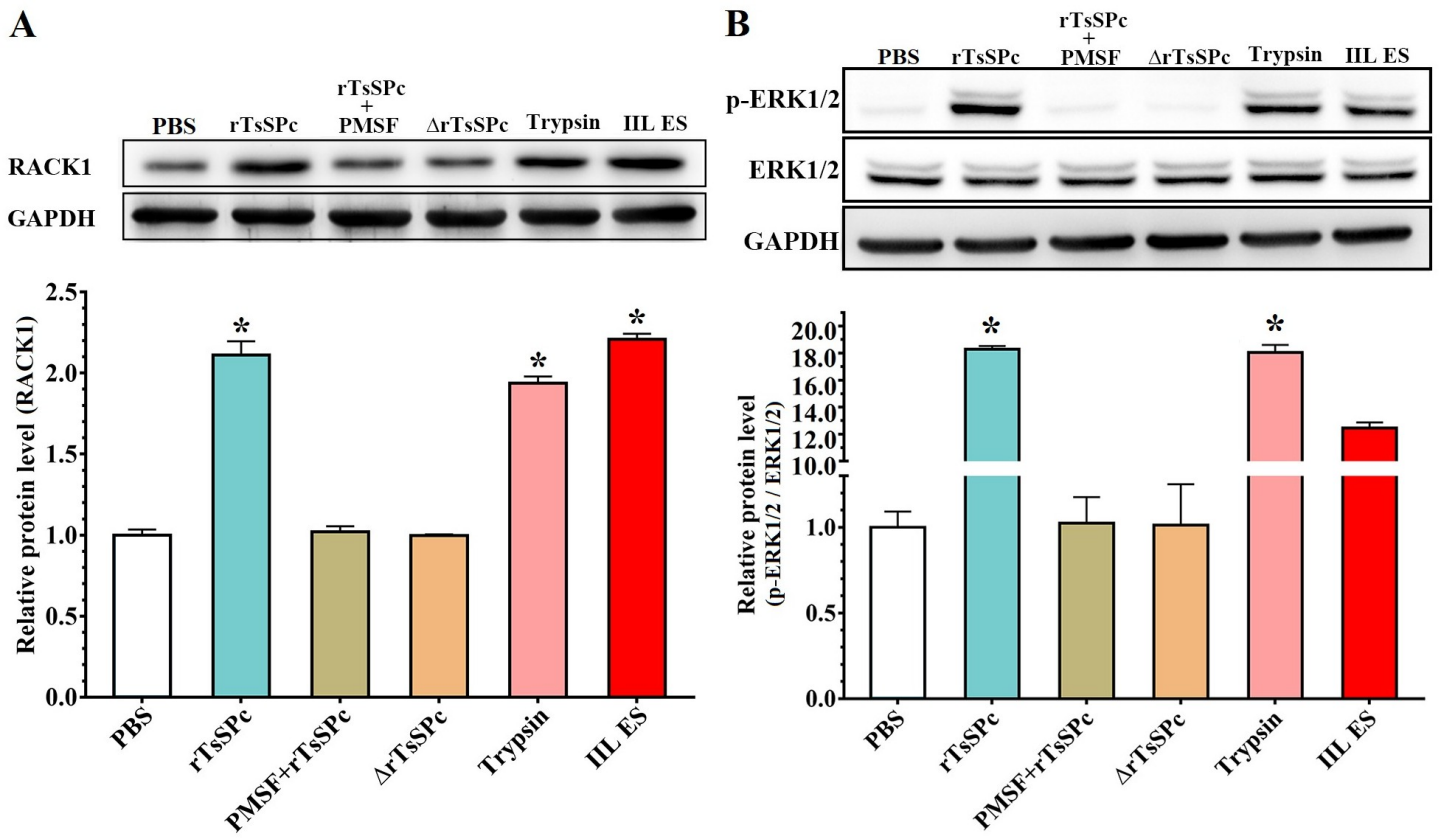
The rTsSPc up-regulated expression of RACK1 and p-ERK1/2 in Caco-2 cells by Western blotting. **A**: rTsSPc up-regulated expression of RACK1. After Caco-2 cells were treated with rTsSPc, trypsin or IIL ES antigens, the RACK1 expression was obviously up-regulated compared to the PBS group. **B**: rTsSPc up-regulated expression of p-ERK1/2. ERK1/2 phosphorylation level in Caco-2 cells treated with rTsSPc was detected by Western blot. Compared to the PBS group, the ERK1/2 MAPK signaling pathway was activated and ERK1/2 phosphorylation level was evidently increased in Caco-2 cells treated with rTsSPc, trypsin and IIL ES antigens. ΔrTsSPc represents the heating inactivated rTsSPc at 100 °C for 5 min, and * in the figure represents *P* < 0.0001 compared to the PBS group. Each experiment was carried out in triplicate.

Western blot results showed that after Caco-2 cells were treated by rTsSPc, expression level of RACK1 receptor was distinctly increased compared to the PBS group. The expression levels of RACK1 receptor in Caco-2 cells treated with rTsSPc, trypsin and IIL ES antigens were increased by 2.11, 1.94 and 2.21 folds respectively compared to the PBS group (*F* = 521.066, *P* < 0.0001). However, expression level of RACK1 was not significantly changed in the rTsSPc+PMSF group (*P* > 0.05) and inactivated rTsSPc group (*P* > 0.05) (Fig 5A). The results indicated that rTsSPc binding to RACK1 evidently up-regulated the RACK1 expression. Furthermore, Western blot results showed that phosphorylated ERK1/2 (p-ERK1/2) level was obviously increased in Caco-2 cells treated with rTsSPc (Fig 5B). Compared to the PBS group, the p-ERK1/2 levels in the ERK1/2 MAPK pathway were increase by 18.29, 18.05, and 12.45 folds in rTsSPc, trypsin and IIL ES antigen group, respectively (*F* = 2099.019, *P* < 0.0001). However, there was no change in pERK1/2 levels in the rTsSPc+PMSF and inactivated rTsSPc group compared to the PBS group (*F* = 0.014, *P* > 0.05). The results suggested that rTsSPc binding to RACK1 significantly increased the expression of RACK1 in Caco-2 monolayer, and consequently activated MAPK pathway and increased the p-ERK1/2 level.

### Caco-2 cell TJs proteins were not hydrolyzed directly by rTsSPc

**Fig 6:**
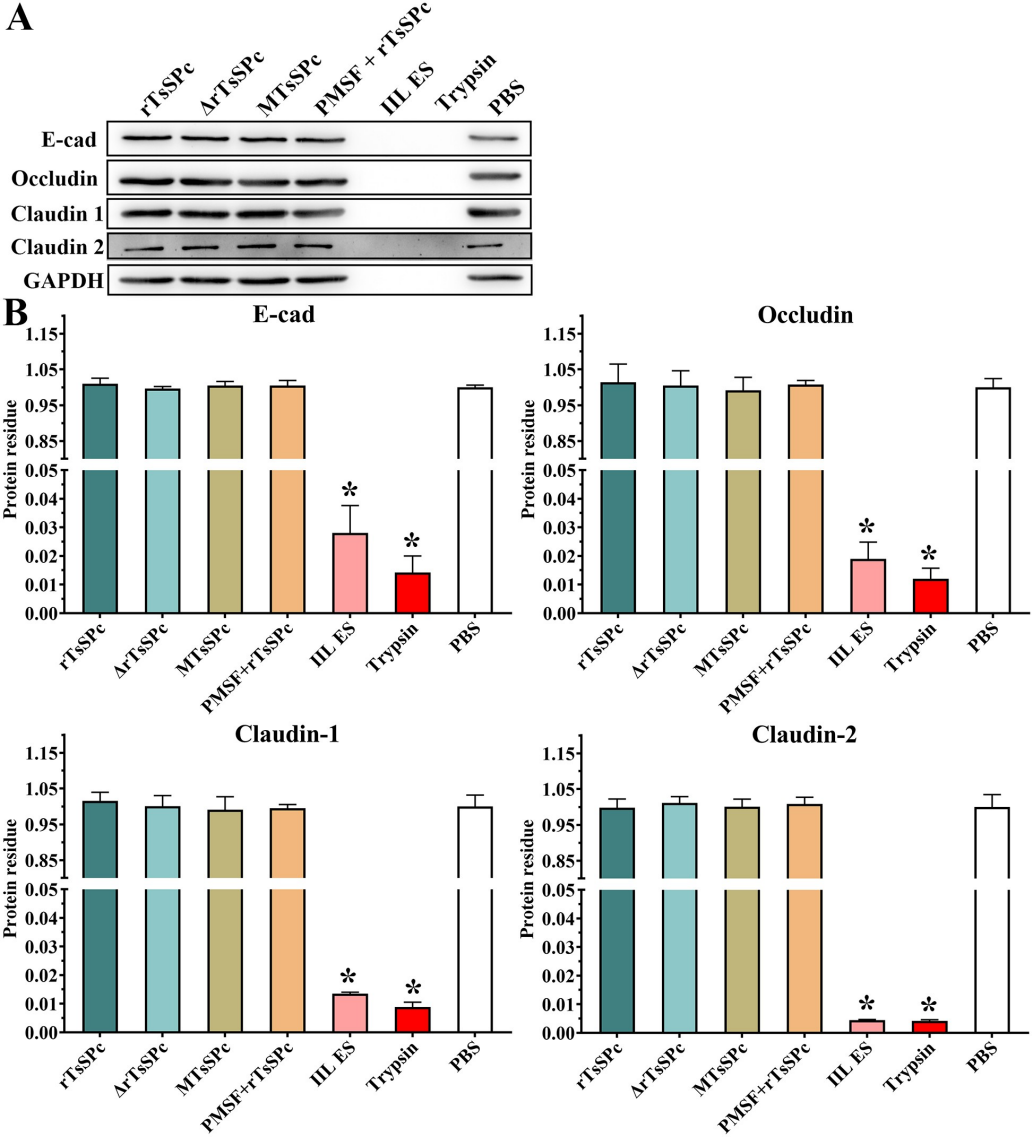
Assessment of direct hydrolysis of Caco-2 monolayer TJs proteins by rTsSPc. **A**: Western blotting of direct hydrolysis of TJs in Caco-2 cell proteins incubated with rTsSPc, direct hydrolysis of E-cad, occludin, claudin-1 and claudin-2 was observed only in Caco-2 cell proteins incubated with IIL ES antigens and trypsin groups. **B**: Quantitative analysis of Western blot results in Figure 6A showed that rTsSPc had no direct degradation role on E-cad, occludin, claudin-1 and claudin-2, relative to the PBS group. ΔrTsSPc represents the heating inactivated rTsSPc, and MTsSPc shows the rTsSPc with enzymatic active site mutation which was prepared in our laboratory. * in the figure represents *P* < 0.0001 compared to the PBS group. Each experiment had three replicates.

Western blot results showed that rTsSPc did not directly hydrolyze the TJs proteins of Caco-2 cells at 37 °C (Fig 6A). After quantification, it was clearly observed that the quantity of E-cad, occludin, claudin-1 and claudin-2 in rTsSPc-incubated Caco-2 cell proteins was not statistically reduced compared to the PBS group (*P* > 0.05) (Fig 6B). However, after Caco-2 cell proteins were incubated with IIL ES antigens, the contents of E-cad, occludin, claudin-1 and claudin-2 was significantly reduced (*F*_E-cad_ = 6201.19, *F*_occludin_ = 764.333, *F*_claudin-1_ = 1240.888, *F*_claudin-2_ = 1697.088, *P* < 0.0001). Treatment with trypsin also reduced the contents of the four TJ proteins (*P* < 0.0001). The results suggested that rTsSPc did not directly hydrolyze the TJs proteins in Caco-2 monolayer, but the natural proteases in IIL ES antigens have the capacity to directly degrade the TJs proteins. The results further confirm that rTsSPc disrupting gut epithelial integrity was not resulted from rTsSPc direct hydrolysis on TJs proteins, while it is likely due to rTsSPc reducing expression of TJs proteins through rTsSPc binding to RACK1 and activating ERK1/2 pathway in Caco-2 cells.

### rTsSPc increased paracellular permeability of Caco-2 monolayer

**Fig 7:**
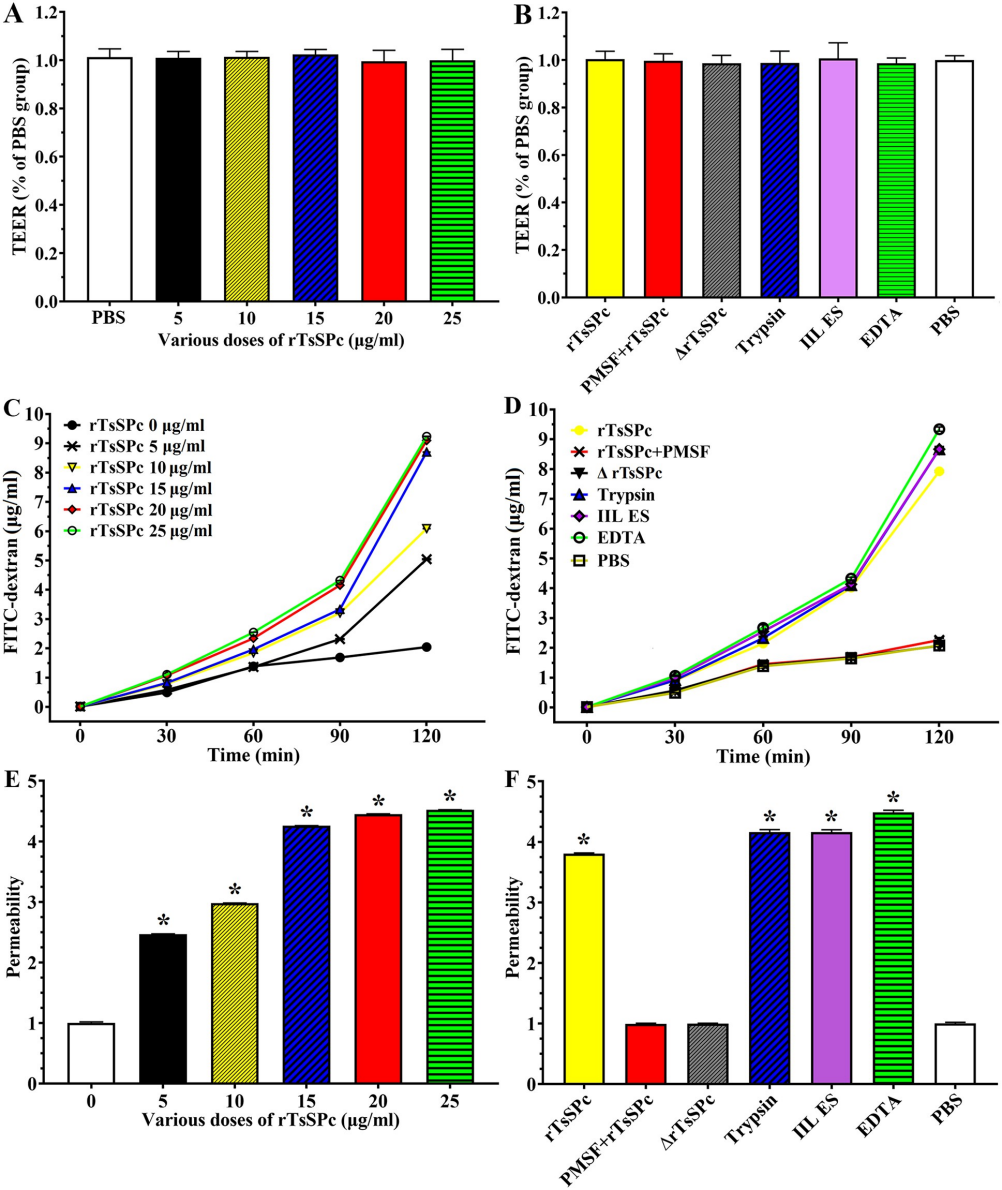
rTsSPc disrupted the integrity of Caco-2 monolayer barrier. When the Caco-2 cells grew to fully the confluence, the integrity of the cell monolayer was assessed by measuring TEER. The apical-to-basolateral flux of FD4TC-dextran through the cell monolayers cultured in Transwell system was measured for 2 h after the addition of dextran in the presence of rTsSPc. Data showed the mean ± SD of three independent experiments. **A**: TEER measure of Caco-2 monolayer before treatment with different doses of rTsSPc. **B**: TEER of Caco-2 monolayer before treatment with rTsSPc and IIL antigens. **C**: Effect of different concentrations of rTsSPc on the permeability of Caco-2 monolayer for diverse incubation times. **D**: Effect of rTsSPc and IIL ES antigens on the permeability of the cell monolayer for diverse incubation times. **E**: Permeability of cell monolayer treated with various doses of rTsSPc for 120 min. **F**: Permeability of cell monolayer treated with rTsSPc and IIL antigens for 120 min. Each experiment had triplicate. ΔrTsSPc represents the heating inactivated rTsSPc at 100 °C for 5 min. *P < 0.0001 relative to the PBS control group.

The TEER assay results showed that the resistance values of different rTsSPc doses of groups (5, 10, 15, 20 and 25 μg/ml) before treatment were 0.998, 1.001, 1.011, 0.984 and 0.998, respectively (*F* = 0.254, *P* > 0.05) (Fig 7A); the resistance values of different experimental groups (rTsSPc, rTsSPc+PMSF, inactivated rTsSPc, trypsin, IIL ES antigens and EDTA) were 1.004, 0.996, 0.986, 0.988, 1.007 and 0.986, respectively prior to treatment, and there was no obvious difference compared to the PBS group (1.000) (*F* = 0.149, *P* > 0.05) (Fig 7B), indicating the integrity of Caco-2 monolayer which can be used for the subsequent permeability assay. The results of paracellular permeability assay showed a positive correlation between the emission values of FITC-dextran and the rTsSPc concentration (0–25 μg/ml) within 2 h (*r* = 0.947, *P* < 0.0001) (Fig 7C). After Caco-2 monolayer was treated, the emissions of FITC-dextran in the rTsSPc, IIL ES antigens, trypsin and EDTA groups were significantly increased every 30 min in the lower chamber samples (*F* = 81039.51, *P* < 0.0001) (Fig 7D). After Caco-2 monolayers were incubated with different rTsSPc concentrations (5, 10, 15, 20 and 25 μg/ml) for 2 h, the permeability of FITC-dextran was 2.47, 2.98, 4.26, 4.45 and 4.52 folds of the PBS group (*F* = 102189.852, *P* < 0.0001) (Fig 7E). After incubation for 2 h, the permeability of FITC-dextran in the chamber of different groups of rTsSPc, rTsSPc+PMSF, inactivated rTsSPc, trypsin, IIL ES antigens and EDTA was 3.81, 0.99, 1.00, 4.16, 4.16 and 4.49 folds of the PBS group (*F* = 12513.112, *P* < 0.0001) (Fig 7F). The results indicated that rTsSPc disrupted the integrity of epithelial cell monolayer, increased the permeability, and reduced the barrier function of gut epithelium.

### Inhibitors and antibody abrogated the rTsSPc down-regulating TJs expression in Caco-2 cells

**Fig 8:**
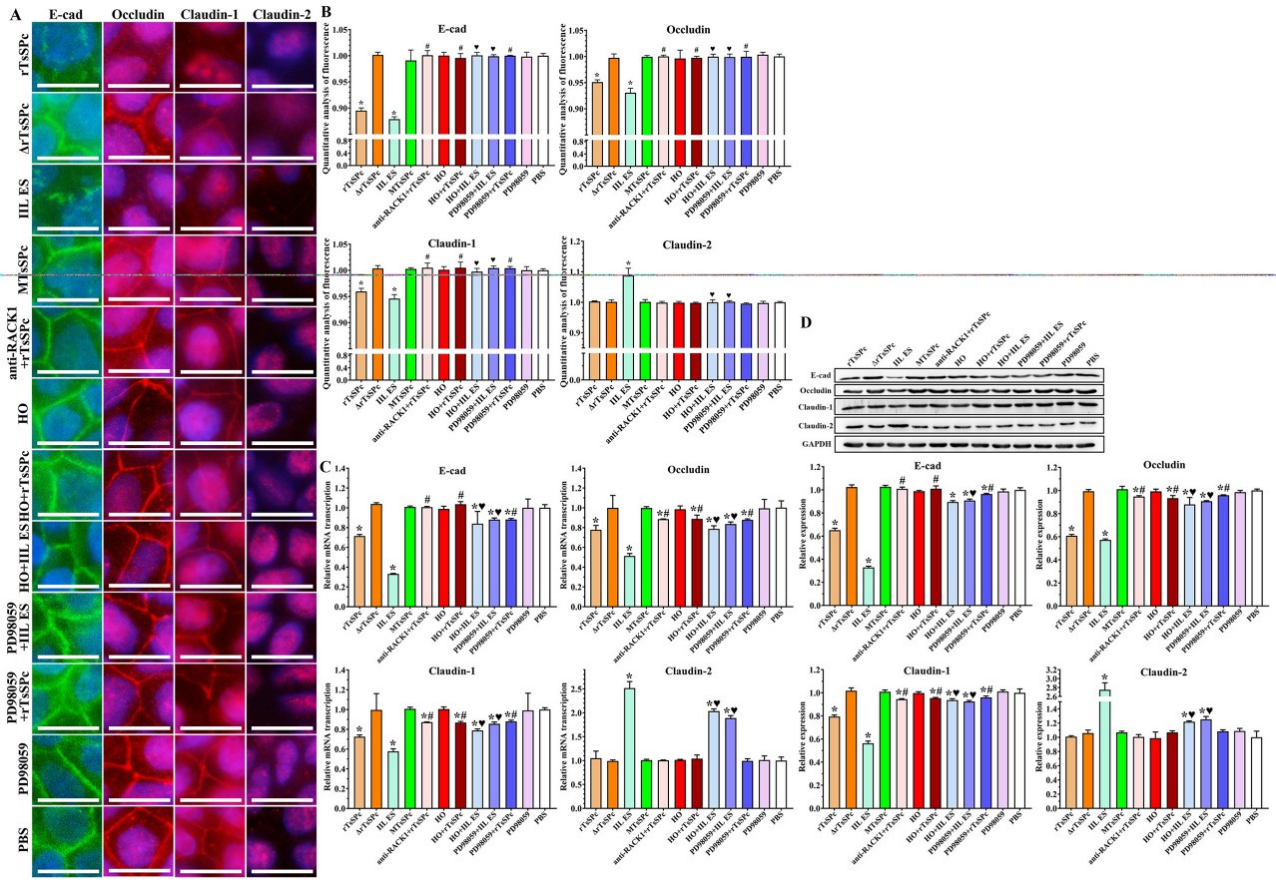
Inhibitors and antibody abrogated the rTsSPc down-regulating TJs expression in Caco-2 cells. **A**: IIFT assay of TJs expression proteins in Caco-2 cells incubated with rTsSPc after pretreatment with inhibitors and anti-RACK1 antibody. Caco-2 monolayer was pre-treated with HO, PD98059 or anti-RACK1 at 37 °C for 24 h, and then incubated with rTsSPc for 2 h. PBS was used as a negative control, IIL ES antigens was used as a positive control. The regulatory effect of rTsSPc on expression of TJs proteins in Caco-2 cells was assessed by IIFT. The treated cells were fixed with 4% paraformaldehyde, permeated with 0.25% Triton X-100 for 10 min, and blocked with 1% BSA. After washing, the cells were probed with primary antibodies against occludin, claudin-1, claudin-2 or E-cad and then incubated with FITC- or Cy3-conjugated secondary antibodies. Cell nuclei were stained blue with DAPI. The cells were observed under a fluorescence microscope (1000×). Scale bars: 10 μm. **B**: Quantitative analysis of the fluorescence. **C**: qPCR analysis of transcription level of TJs protein E-cad, occludin, claudin-1, and claudin-2 genes relative to the PBS group. **D**: Western blotting of expression level of TJs proteins in Caco-2 cells incubated with rTsSPc after pretreatment with HO, PD98059, and anti-RACK1 antibody. Expression level of E-cad, occludin, claudin-1 and claudin-2 in Caco-2 cells was compared to the PBS group, only rTsSPc or IIL ES antigen alone group. Each experiment was performed in triplicate. ΔrTsSPc represents the heating inactivated rTsSPc, MTsSPc shows mutant rTsSPc. *P < 0.05 compared to the PBS group, ^#^*P* < 0.05 compared to the rTsSPc group; ^♥^*P* < 0.05 relative to the IIL ES antigen group.

The IIFT results revealed that after Caco-2 cells were incubated with rTsSPc and IIL ES antigens, the expression levels of occludin, claudin-1 and E-cad were significantly decreased, as demonstrated that the continuous fluorescence staining around the cells was disappeared (Fig 8A). However, after the cells were pre-treated with RACK1 inhibitor HO, anti-RACK1 antibody, or ERK1/2 pathway inhibitor PD98059, expression levels of occludin, claudin-1 and E-cad were restored, namely the two inhibitors and anti-RACK1 antibody suppressed and abolished the rTsSPc down-regulating on expression of occludin, claudin-1 and E-cad. Moreover, expression level of claudin-2 was partially increased only after the cells incubated with IIL ES antigens; claudin-2 expression had no significant change in the rTsSPc group and other groups. The semi-quantitative IIFT results showed that after Caco-2 monolayer was pre-treated with two inhibitors and anti-RACK1 antibody, the expression of occludin, claudin-1 and E-cad was obviously regained and increased (Fig 8B). Occludin content in the rTsSPc and IIL ES antigen group was decreased by 0.049 and 0.069 folds, respectively, compared to the PBS group (*F* = 30.430, *P* < 0.0001); the occludin in the HO+rTsSPc, PD98059+rTsSPc, and anti-RACK1+rTsSPc group was increased by 0.046, 0.049 and 0.049 folds respectively, compared to the rTsSPc group (*F* = 47.664, *P* < 0.0001). Additionally, compared to the PBS group, claudin-1 in the rTsSPc and IIL ES antigen group was decreased by 0.040 and 0.054 folds (*F* = 29.126, *P* < 0.0001); whereas claudin-1 in the HO+rTsSPc, PD98059+rTsSPc and anti-RACK1+rTsSPc group was increased by 0.045, 0.044 and 0.045 folds compared to the rTsSPc group (*F* = 25.936, *P* < 0.0001). The E-cad in the rTsSPc and IIL ES antigen group was decreased by 0.105 and 0.121 folds compared to the PBS group (*F* = 86.567, *P* < 0.0001); but E-cad expression in the HO+rTsSPc, PD98059+rTsSPc and anti-RACK1+rTsSPc group was respectively increased by 0.101, 0.105 and 0.106 folds relative to the rTsSPc group (*F* = 179.906, *P* < 0.0001). However, compared to the PBS group, claudin-2 expression level was increased by 0.088 folds only in the IIL ES antigen group (*F* = 28.457, *P* < 0.0001), and there was no statistically significant difference between the rTsSPc group and other groups (*P* > 0.05). The IIFT results indicated that the rTsSPc down-regulating the TJs (occludin, claudin-1 and E-cad) expression in Caco-2 cells was significantly abrogated and blocked by the inhibitors and anti-RACK1 antibody, and further suggested that rTsSPc regulated expression of TJs proteins in Caco-2 cells through binding to RACK1 receptor and activating the ERK1/2 pathway.

qPCR results showed that after Caco-2 cells were incubated with rTsSPc, the E-cad transcription level was evidently reduced compared to the PBS group; but when the cells were pre-treated with HO, PD98059 and anti-RACK1 antibody, the E-cad transcription level was evidently restored compared to the rTsSPc group (*F* = 83.235, *P* < 0.0001), indicating that inhibitors and antibody suppressed and abrogated the rTsSPc down-regulating E-cad transcription level (Fig 8C). rTsSPc down-regulated the occludin transcription levels, but HO, PD98059 and anti-RACK1 antibody regained the occludin transcription levels (*F* = 9.390, *P* < 0.01). The claudin-1 transcription level was obviously decreased in the cells incubated with rTsSPc, but when the cells were pre-treated with HO, PD98059 and anti-RACK1 antibody, the claudin-1 transcription level was restored (*F* = 123.17, *P* < 0.0001). Compared to the PBS group, transcription level of claudin-2 was not statistically changed in the cells incubated with rTsSPc or rTsSPc+inhibitors (*P* > 0.05). The results demonstrated that RACK1 receptor and ERK1/2 pathway inhibitor, and anti-RACK1 antibody obviously blocked the rTsSPc binding to RACK1 receptor and intercepted the ERK1/2 pathway in Caco-2 cells, and abolished the rTsSPc down-regulating role on TJs expression. The results further verified that rTsSPc down-regulated the TJs protein expression by binding to RACK1 in Caco-2 cells and activating the ERK1/2 pathway.

Western blot results showed that after Caco-2 cells were pretreated with RACK1 receptor inhibitor HO, ERK1/2 pathway inhibitor PD98059, and anti-RACK1 antibody, then incubated with rTsSPc, the two inhibitors and antibody suppressed and abrogated the rTsSPc down-regulating TJs expression in Caco-2 cells (Fig 8D). In three groups pretreated with HO, PD98059, and anti-RACK1 antibody, E-cad expression level in Caco-2 cells was evidently restored after incubated with rTsSPc compared to the only rTsSPc group (*F* = 302.741, *P* < 0.0001). After pretreatment with HO, PD98059, and anti-RACK1 antibody, occludin expression level in Caco-2 cells incubated with rTsSPc was obviously recovered and increased compared to the alone rTsSPc group in which the cells were not pretreated with inhibitors and antibody (*F* = 466.696, *P* < 0.0001). The claudin-1 expression level in two inhibitor groups and one antibody groups were distinctly higher than the rTsSPc alone group (*F* = 200.475, *P* < 0.0001). Moreover, rTsSPc, inhibitors and RACK1-specific antibody had no evident effects on claudin-2 expression level in Caco-2 cells. The results indicated that the rTsSPc down-regulating expression of E-cad, occludin and claudin-1 in Caco-2 cells was evidently suppressed and abolished by the two inhibitors and anti-RACK1 antibody. The results further suggested that rTsSPc binding to RACK1 receptor in Caco-2 cells activated ERK1/2 pathway, consequently reduced the expression of TJs proteins and damaged the gut epithelial cell barrier, and might facilitate the *T*. *spiralis* IIL invasion of host gut mucosa.

### Inhibitors suppressed rTsSPc inducing RACK1 expression and ERK1/2 pathway activation

**Fig 9:**
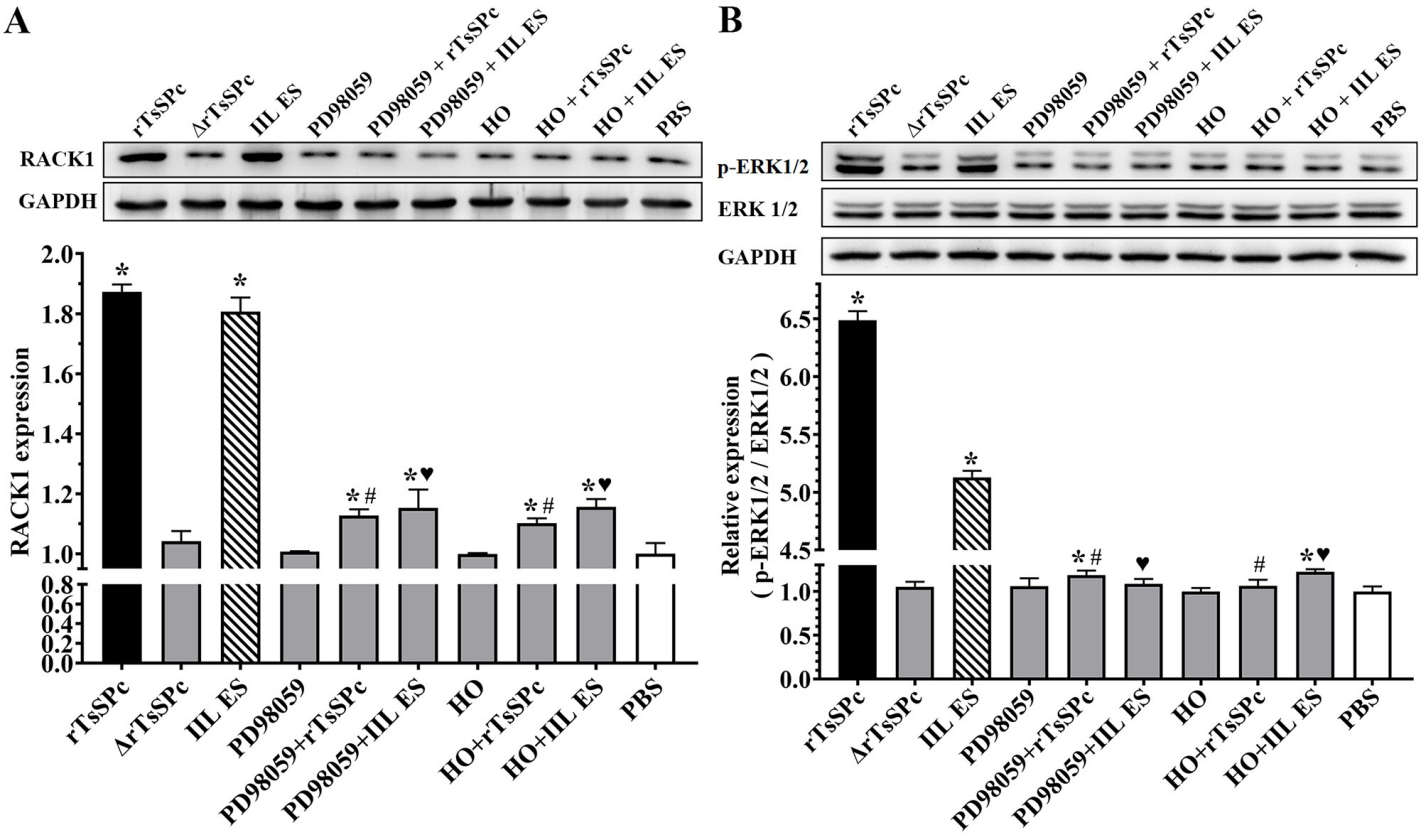
RACK1 receptor and ERK1/2 pathway inhibitors abrogated rTsSPc activating ERK1/2 pathway. Caco-2 monolayer was pretreated with two inhibitors HO and PD98059, and then incubated with rTsSPc, the ERK1/2 pathway was not activated in inhibitors-pretreated Caco-2 cells. **A**: Western blotting analysis of RACK1 expression level in Caco-2 cells. **B**: Western blotting analysis of ERK1/2 expression level in Caco-2 cells. Quantitative analysis of protein bands in Western blot was performed by using ImageJ software. ΔrTsSPc represents the heating inactivated rTsSPc. Each experiment was performed in triplicate. * in the figure represents *P* < 0.05 compared to the PBS group. ^#^*P* < 0.05 compared to the rTsSPc group; ^♥^*P* < 0.05 relative to the IIL ES antigen group.

The Western blot results showed that when Caco-2 cells were incubated with rTsSPc, the level of RACK1 and p-ERK1/2 was significantly elevated compare to the PBS group; but after the Caco-2 cells were pretreated with RACK1 receptor inhibitor HO and ERK1/2 pathway inhibitor PD98059, then incubated with rTsSPc, the expression levels of RACK1 and p-ERK1/2 was dropped (Fig 9A and 9B). Quantitative analysis of protein bands in Western blot showed that the RACK1 levels in the HO+rTsSPc and PD98059+rTsSPc group were significantly lower than those in the rTsSPc group alone (*F* = 754.238, *P* < 0.0001). The p-ERK1/2 level in two inhibitor groups (HO + rTsSPc, and PD98059 + rTsSPc) was obviously lower than the rTsSPc alone group (*F* = 8263.178, *P* < 0.0001). The results showed that the two inhibitors restrained and abolished the rTsSPc inducing expression of RACK1 and p-ERK1/2 and activating ERK1/2 pathway, validated that rTsSPc binding with RACK1 receptor in Caco-2 cells activated ERK1/2 signaling pathway.

### Inhibitors and antibody suppressed larval intrusion of Caco-2 monolayer and reduced intestinal worm burdens in infected mice

**Fig 10:**
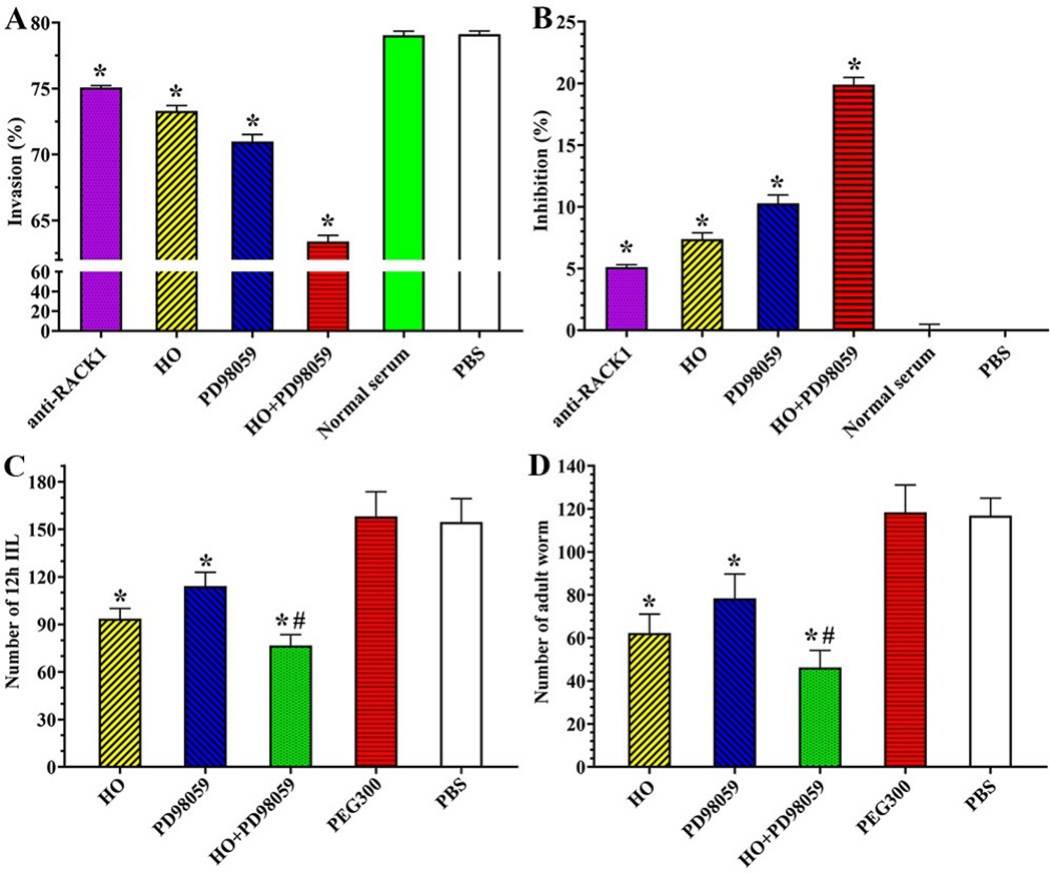
Inhibitors and anti-RACK1 antibody suppressed larval invasion and reduced intestinal worm burden in infected mice. **A**: Inhibitors and anti-RACK1 antibody inhibited larval invasion of Caco-2 cells. The muscle larvae were first activated into the IIL with 5% porcine bile at 37 °C for 2 h, and then were added onto the Caco-2 monolayer pretreated with inhibitors or anti-RACK1 antibody and incubated for 24 h. The larval invasion was observed under a microscope. **B**: The data were expressed as the inhibition (%) normalized to the PBS control group. HO and PD98059 also reduced intestinal IIL and adult burden in infected mice at 12 hours and 5 days after infection. **C**: 12 h IIL burden (n = 10); **D**: Intestinal adult worm burden (n = 10). Each experiment was carried out in triplicate, and the data of each group were represented as mean ± standard deviation. *P < 0.05 relative to the PBS group, ^#^*P* < 0.05 relative to the only HO or PD98059 alone group.

When Caco-2 cells were pre-treated with anti-RACK1 antibody, RACK1 receptor inhibitor HO, ERK1/2 pathway inhibitor PD98059, or HO+PD98059 for 2 h, the IIL invasion rate of Caco-2 monolayer were 75.09, 73.31, 70.99 and 63.39%, respectively. The invasion rate of anti-RACK1 antibody, HO, PD98059 and PD98059 + HO group was significantly lower than the PBS group (*F* = 746.388, *P* < 0.0001) (Fig 10A). Namely, the inhibition rate of anti-RACK1 antibody, HO, PD98059 and PD98059 + HO on larval invasion were 5.12, 7.37, 10.29 and 19.89% compared to the PBS group (*F* = 746.388, *P* < 0.0001) (Fig 10B). The results showed that RACK1 receptor and ERK1/2 pathway inhibitors, and anti-RACK1 antibody obviously impeded larval invasion of Caco-2 monolayer, hindered the binding of rTsSPc with RACK1 receptor and intercepted the ERK1/2 pathway in Caco-2 cells, further confirmed that rTsSPc binding to RACK1 in Caco-2 cells activated the ERK1/2 pathway, down-regulated the TJs expression, disrupted gut epithelial barrier integrity, consequently mediated the larval invasion of gut mucosa.

All infected mice were euthanized at 12 hpi and 5 dpi. Intestinal IIL and adult worms were recovered and numbered. The results showed that compared to the PBS group, the IIL burden of HO, PD98059 and HO+PD98059 groups was decreased by 39.46 (*F* = 78.626, *P* < 0.0001), 26.13 (*F* = 33.353, *P* = 0.003) and 50.45% (*F* = 125.387, *P* < 0.0001), and the IIL burden in the solvent PEG300 group had no obvious change compared to the PBS group (*P* > 0.05). The IIL burden of the HO+PD98059 group was decreased by 11.00 or 24.32% compared to the only HO or only PD98059 group (*F*_HO_ = 32.238, *F*_PD98059_ = 114.386, *P* < 0.0001) (Fig 10C). At 5 dpi, compared to the PBS group, the adult burden of HO, PD98059 and HO+PD98059 groups was decreased by 46.71, 32.93 and 60.39%, respectively (*F*_HO_ = 100.268, *F*_PD98059_ = 43.11, *F*_HO+PD98059_ = 175.241, *P* < 0.001) (Fig 10D). The adult burden of the HO+PD98059 group was decreased by 13.69 and 27.46% compared to the only HO and only PD98059 group, and it was significantly lower than the group using only HO or PD98059 alone (*F*_HO_ = 18.225, *F*_PD98059_ = 53.528, *P* < 0.0001), suggesting that HO+PD98059 had synergistic suppressive effect on larval invasion and reducing enteral adult burden. The results indicated that the RACK1 inhibitor HO and ERK1/2 inhibitor PD98059 significantly inhibited the larval invasion and development in host intestine, and the inhibitory effect of HO+PD98059 was higher than the single HO and PD98059 alone.

### Inhibitors suppressed expression of RACK1 and p-ERK1/2 in infected mice

**Fig 11:**
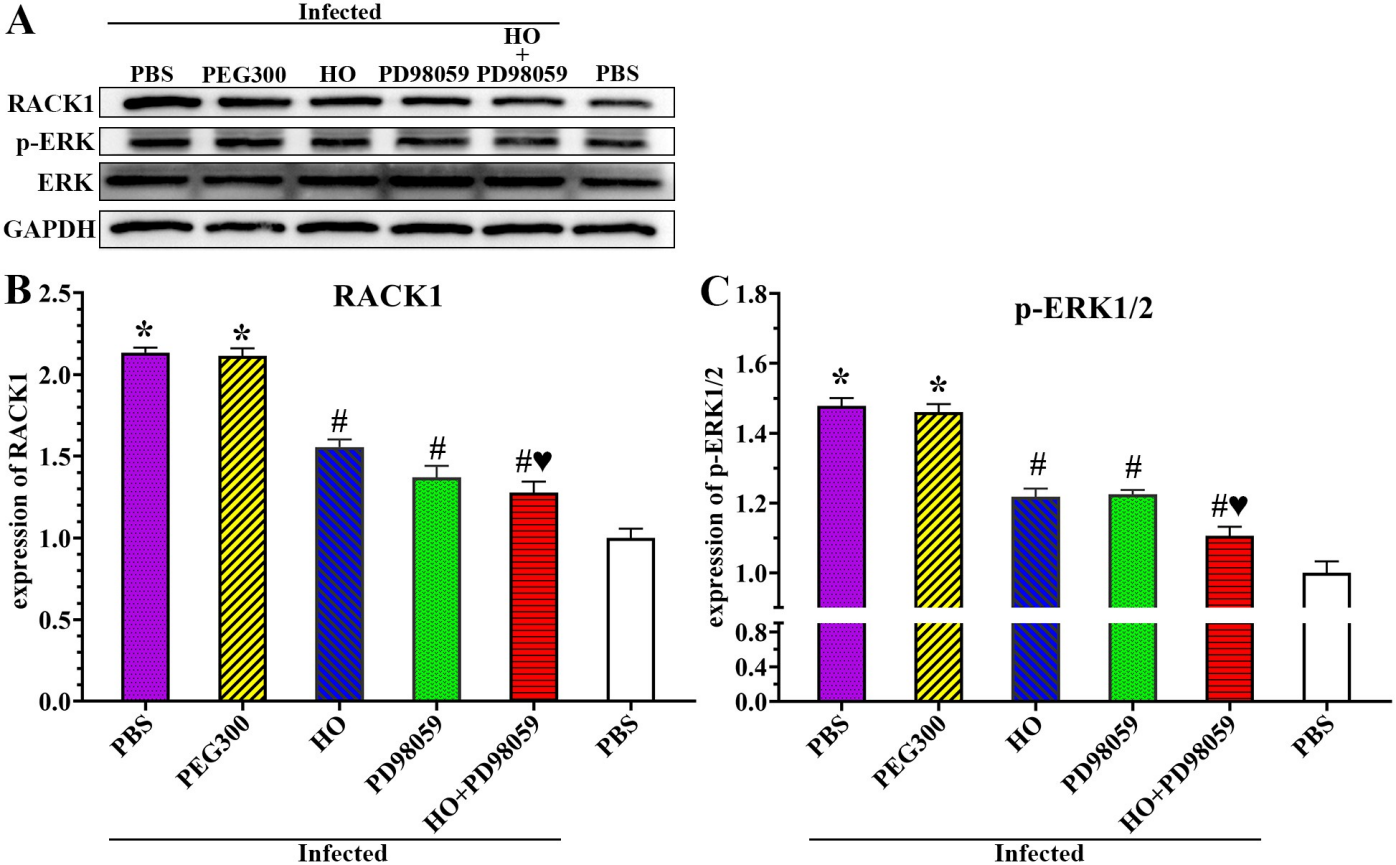
HO and PD98059 inhibited up-regulation of RACK1 and p-ERK1/2 caused by *T*. *spiralis* infection. **A**: Western blotting of expression of RACK1 and p-ERK in infected mouse intestine. **B**: HO and PD98059 inhibited the RACK1 expression in infected mice. **C**: HO and PD98059 decreased up-regulation of p-ERK1/2 in infected mice. Each test had three replicates. *P < 0.0001 compared to the uninfected PBS group; ^#^*P* < 0.001 relative to the infected PBS group; ^♥^*P* < 0.05 compared to the only HO or PD98059 alone group.

At 5 dpi, intestinal tissues were collected from *T*. *spiralis*-infected mice pretreated with HO and PD98059, expression levels of RACK1 receptor and p-ERK1/2 were assessed by western blot. The results showed that expression levels of RACK1 and p-ERK1/2 in the infected PBS group were significantly higher than those in the uninfected PBS group (*F*_RACK1_ = 599.374, *F*_p-ERK1/2_ = 318.730, *P* < 0.0001) (Fig 11A). The RACK1 expression level of HO, PD98059 and HO+PD98059 group was decreased by 0.58, 0.76 and 0.86 folds, respectively, compared to the infected PBS group (*F*_HO_ = 173.438, *P* < 0.0001; *F*_PD98059_ = 51.309, *F*_HO+PD98059_ = 30.411, *P* < 0.05) (Fig 11B). The p-ERK1/2 expression levels in HO, PD98059, and HO+PD98059 groups were reduced by 0.26, 0.25 and 0.37 folds respectively relative to the infected PBS mice (*F*_HO_ = 173.438, *F*_PD98059_ = 295.945, *F*_HO+PD98059_ = 354.055, *P* < 0.001) (Fig 11C). The expression level of RACK1 and p-ERK1/2 in the HO+PD98059 group were evidently lower than those in the individual HO or PD98059 alone group (*F*_RACK1_ = 15.859, *F*_p-ERK1/2_ = 28.340, *P* < 0.05). The results showed that *T*. *spiralis* infection increased significantly expression level of RACK1 and p-ERK1/2 in gut epithelium, whereas RACK1 receptor inhibitor HO and ERK1/2 pathway inhibitor PD98059 declined and abrogated the *T*. *spiralis* infection-increased expression level of RACK1 and p-ERK1/2. The results further verified that *T*. *spiralis* infection increased RACK1 expression and activated ERK1/2 pathway.

### Inhibitors up-regulated the expression of gut TJs proteins in infected mice

**Fig 12:**
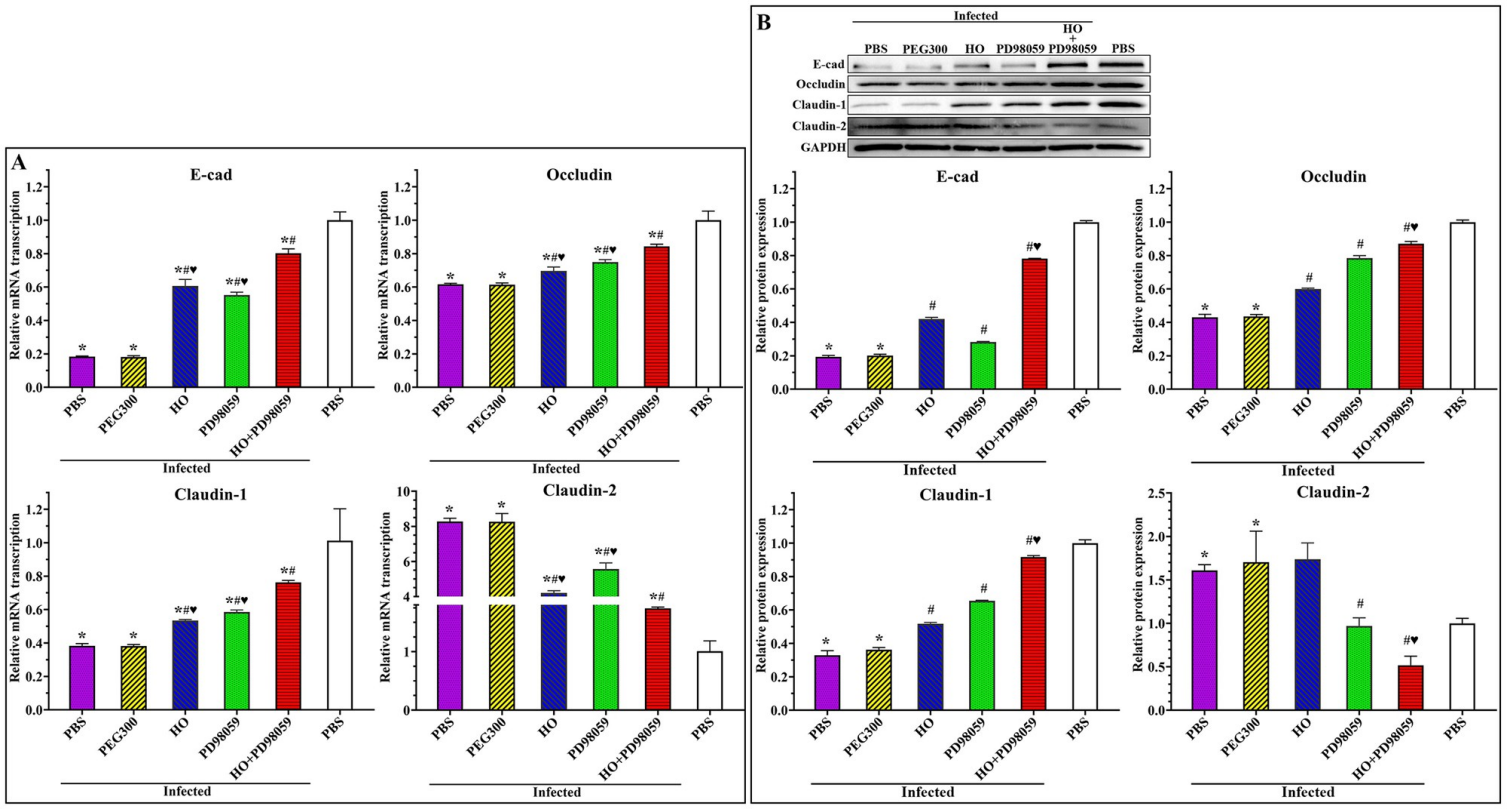
HO and PD98059 up-regulated the transcription and expression of gut TJs proteins in infected mice. **A**: qPCR analysis of transcription level of TJs proteins in infected mice pretreated by HO and PD98059. **B**: Western blotting of TJs protein expression in infected mice pretreated by HO and PD98059. HO and PD98059 pretreatment increased the expression levels of TJs (E-cad, occludin and claudin-1) and decreased claudin-2 expression in *T*. *spiralis*-infected murine intestine. Each test had three replicates. * *P* < 0.05 compared with the uninfected PBS group; # *P* < 0.05 compared with the infected PBS group; ^♥^*P* < 0.05 compared to the only HO or PD98059 alone group.

The qPCR results showed that the transcription levels of E-cad, occludin and claudin-1 in the infected PBS group were significantly decreased relative to the uninfected PBS group (blank PBS control group); the claudin-2 transcription level was evidently increased relative to uninfected PBS group. The transcription level of E-cad, occludin, and claudin-1 in the HO, PD98059 and HO+PD98059 groups was significantly increased compared to the infected PBS group, while claudin-2 transcription was decreased. The transcription levels of E-cad in the PBS infected groups was reduced by 0.82 folds relative to the uninfected PBS group (*F* = 807.258, *P* < 0.0001); The E-cad transcription level of the HO, PD98059 and HO+PD98059 groups was increased by 2.30, 2.01 and 3.37 folds, respectively, compared to the PBS infected group (*F*_HO_ = 349.787, *F*_PD98059_ = 1323.241, *F*_HO+PD98059_ = 1654.568, *P* < 0.0001) (Fig 12A). The occludin transcription levels of PBS infected group was decreased by 0.38 folds (*F* = 145.729, *P* < 0.0001) compared to the PBS uninfected group; the occludin transcription level of the HO, PD98059 and HO+PD98059 groups was increased by 0.13, 0.22 and 0.37 folds respectively, compared to the PBS infected group (*F*_HO_ = 31.123, *F*_PD98059_ = 191.174, *F*_HO+PD98059_ = 652.554, *P* < 0.0001). Moreover, the claudin-1 transcription levels of PBS infected group was decreased by 0.62 folds compared to the uninfected PBS group (*F* = 32.298, *P* < 0.01); the claudin-1 transcription levels of the HO, PD98059 and HO+PD98059 groups was elevated by 0.40, 0.53 and 0.99 folds respectively, compared to the PBS infected group (*F*_HO_ = 3116.794, *F*_PD98059_ = 380.249, *F*_HO+PD98059_ = 1297.17, *P* < 0.0001). However, the claudin-2 transcription levels of PBS infected group was increased by 7.20 folds compared to the uninfected PBS group and (*F* = 558.032, *P* < 0.0001); and the claudin-2 transcription levels of HO, PD98059, and HO+PD98059 groups was decreased by a 4.08, 2.70 and 6.54 folds respectively, compared to the PBS infected group (*F*_HO_ = 969.295, *F*_PD98059_ = 144.490, *F*_HO+PD98059_ = 3734.112, *P* < 0.0001). The transcription level of E-cad, occludin, and claudin-1 in the HO+PD98059 group was evidently higher than the individual HO or PD98059 alone group (*F*_E-cad_ = 62.573, *F*_occludin_ = 49.145, *F*_claudin-1_ = 389.947, *P* < 0.0001), while the claudin-2 transcription levels of the HO+PD98059 group was significantly lower than the single HO or PD98059 alone group (*F* = 250.255, *P* < 0.0001). The results demonstrated that HO and PD98059 increased and abrogated the reduced transcription of TJs (E-cad, occludin and claudin-1) caused by *T*. *spiralis* infection; and reduced and abolished the elevated transcription of claudin-2 caused by *T*. *spiralis* infection, further suggesting that RACK1 receptor and ERK1/2 pathway in gut epithelium play an important role for *T*. *spiralis* invasion at early stage of infection.

Western blot results showed that HO and PD98059 significantly upregulated the expression level of TJs protein in infected murine intestine (Fig 12B). The expression levels of E-cad, occludin, and claudin-1 in the infected PBS group were decreased by 0.81, 0.57 and 0.67 folds, respectively, compared to the uninfected PBS group (*F*_E-cad_ = 8295.546, *F*_occludin_ = 1569.94, *F*_claudin-1_ = 1005.481, *P* < 0.0001). But, the E-cad expression of the HO, PD98059 and HO+PD98059 groups was increased by 0.28, 0.11 and 0.73 fold, respectively, compared to the infected PBS group (*F*_HO_ = 953.941, *F*_PD98059_ = 272.600, *F*_HO+PD98059_ = 13253.930, *P* < 0.0001). The E-cad expression of HO+PD98059 group was increased by 0.61 and 0.85 folds relative to the only HO or PD98059 groups (*F* = 6101.772, *P* < 0.0001). Compared to the infected PBS group, occludin expression in the HO, PD98059, and HO+PD98059 groups was increased by 0.30, 0.62 and 0.77 folds, respectively (*F*_HO_ = 239.854, *F*_PD98059_ = 678.491, *F*_HO+PD98059_ = 112.0054, *P* < 0.0001). The occludin expression of HO+PD98059 group was increased by 0.62 and 0.20 folds compared to the single HO or PD98059 alone group (*F* = 378.063, *P* < 0.0001). The claudin-1 expression in the HO, PD98059, and HO+PD98059 groups was increased by 0.28, 0.49, and 0.88 folds compared to the infected PBS group (*F*_HO_ = 135.296, *F*_PD98059_ = 438.480, *F*_HO+PD98059_ = 1324.647, *P* < 0.0001). The claudin-1 expression of HO+PD98059 group was increased by 0.68 and 0.45 folds relative the only HO or PD98059 groups (*F* = 2737.539, *P* < 0.0001). However, claudin-2 expression in the infected PBS group was increased by 0.61 folds compared to the uninfected PBS group (*F* = 9.737, *P* < 0.05). At the same time, compared to the infected PBS group, claudin-2 expression in PD98059 and HO+PD98059 groups was decreased by 1.05 and 1.79 folds (*F*_PD98059_ = 91.109, *P* < 0.01; *F*_HO+PD98059_ = 230.198, *P* < 0.0001), the claudin-2 expression in HO+PD98059 group was decreased by 0.41 relative to the only PD98059 group (*F* = 61.626, *P* < 0.0001). The transcript and expression levels of TJs (E-cad, occludin, claudin-1) was consistent. The results indicated that RACK1 receptor inhibitor HO, ERK1/2 pathway inhibitor PD98059 abrogated the down-regulated expression of TJs (E-cad, occludin and claudin-1) and abolished up-regulated expression of claudin-2 caused by *T*. *spiralis* infection, further suggesting that TsSPc binding to RACK1 in gut epithelium at early stage of *T*. *spiralis* infection activated ERK1/2 pathway, down-regulated the TJs expression, disrupted gut epithelial barrier integrity, consequently mediated the gut epithelium invasion.

### Inhibitors reduced intestinal inflammation and permeability in infected mice

**Fig 13:**
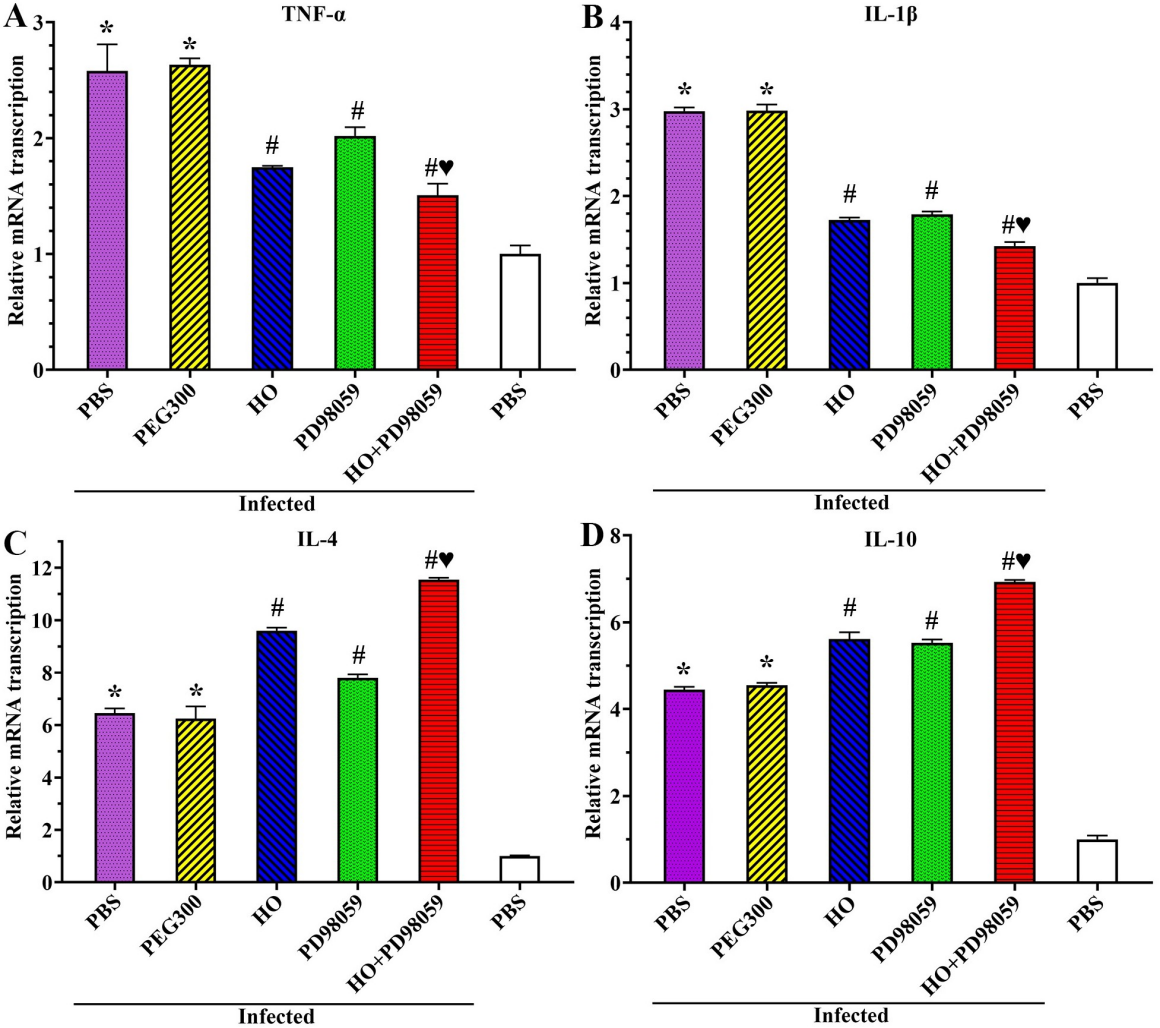
HO and PD98059 alleviated intestinal inflammation caused by *T*. *spiralis* infection. **A** and **B**: HO and PD98059 inhibited and abrogated the elevated transcription level of pro-inflammatory cytokines (TNF-α and IL-1β) caused by *T*. *spiralis* infection. **C** and **D**: HO and PD98059 up-regulated further the transcription level of anti-inflammatory cytokines (IL-4 and IL-10) caused by *T*. *spiralis* infection. Each experiment was carried out in triplicate. * *P* < 0.05 compared to the uninfected PBS group; ^#^ *P* < 0.05 compared to the infected PBS group; ^♥^*P* < 0.05 compared to the only HO or PD98059 alone group.

Transcription levels of inflammatory cytokines in infected murine intestines were assessed by qPCR. qPCR results showed that transcription level of inflammatory cytokines (TNF-α, IL-1β, IL-4 and IL-10) in infected PBS group was prominently increased compared to the uninfected PBS group (*F*_TNF-α_ = 127.11, *F*_IL-1β_ = 1164.57, *F*_IL-4_ = 337.987, *F*_IL-10_ = 2439.694, *P* < 0.0001) (Fig 13). However, after the inhibitors were used, transcription levels of TNF-α and IL-1β in the HO, PD98059 and HO+PD98059 groups were significantly lower than the infected PBS group (TNF-α: *F*_HO_ = 39.269; *F*_PD98059_ = 16.132, *F*_HO+PD98059_ = 55.031, *P* < 0.05. IL-1β: *F*_HO_ = 1760.206, *F*_PD98059_ = 1488.003, *F*_HO+PD98059_ = 1904.720, *P* < 0.0001). The transcription levels of IL-4 and IL-10 in the HO, PD98059 and HO+PD98059 groups were significantly higher than the infected PBS group (IL-4: *F*_HO_ = 628.577, *F*_PD98059_ = 106.593, *F*_HO+PD98059_ = 1947.938, *P* < 0.0001. IL-10: *F*_HO_ = 138.281, *F*_PD98059_ = 353.490, *F*_HO+PD98059_ = 2833.948, *P* < 0.0001). Additionally, the transcription level of TNF-α and IL-1β in the HO+PD98059 group was significantly lower than the individual HO or PD98059 alone group (*F*_TNF-α_ = 8.783, *F*_IL-1β_ = 8.000, *P* < 0.05), while the IL-4 and IL-10 transcription level of the HO+PD98059 group was significantly higher than the single HO or PD98059 group (*F*_IL-4_ = 2977.798, *F*_IL-10_ = 968.241, *P* < 0.0001). The results further indicated that HO and PD98059 inhibited the *T*. *spiralis* invasion of intestinal mucosa, reduced the expression of pro-inflammatory cytokines, and up-regulated the expression of anti-inflammatory cytokines, therefore, meliorated intestinal inflammation caused by *T*. *spiralis* infection.

**Fig 14:**
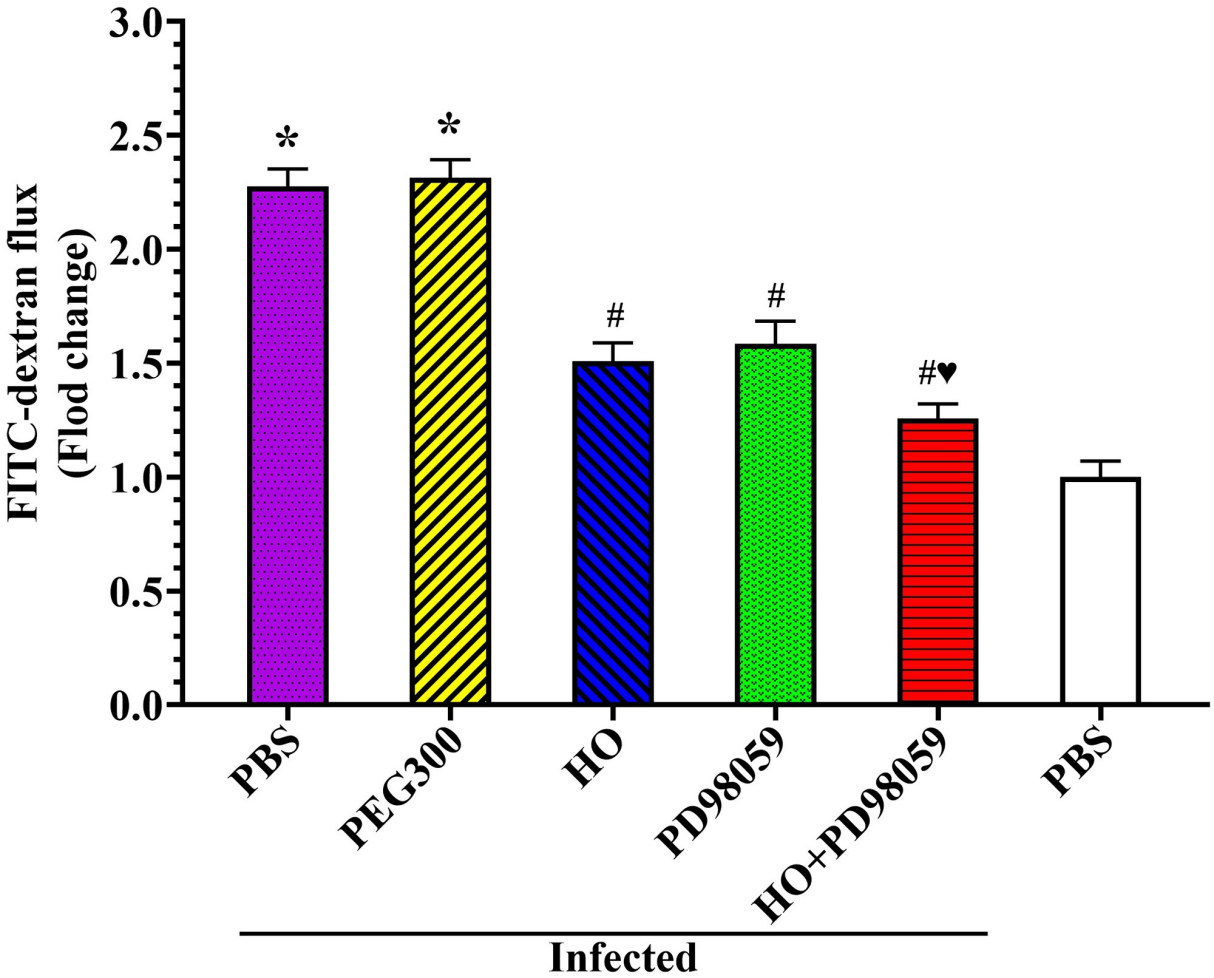
HO and PD98059 suppressed and abrogated the increased intestinal permeability caused by *T*. *spiralis* infection. HO and PD98059 reduced intestinal FD-4 flow. Each assay had six replicates in each group, * *P* < 0.0001 relative to the uninfected PBS group; ^#^*P* < 0.0001 in comparison with infected PBS group. ^♥^*P* <0.0001 in comparison with the only HO or PD98059 alone group.

When mice were first pre-treated mice with HO, PD98059 and HO+PD98059, and then challenged with *T*. *spiralis*, intestinal permeability assay showed the HO and PD98059 significantly inhibited and decreased the elevated intestinal permeability caused by *T*. *spiralis* infection (Fig 14). At 4 hours after FD-4 administration at 5 dpi, the FD-4 flux in infected PBS group was increased by 2.27 folds compared to the uninfected PBS group (*F* = 889.514, *P* < 0.0001), suggesting that *T*. *spiralis* infection evidently increased the intestinal permeability. But when the inhibitors HO or PD98059 were administered, the intestinal permeability of infected mice was prominently decreased relative to the infected PBS group. The FD-4 flux of HO, PD98059 and HO+PD98059 groups was decreased by 0.34 (*F* = 284.656, *P* < 0.0001), 0.30 (*F* = 176.759, *P* < 0.0001) and 0.45 folds (*F* = 613.970, *P* < 0.0001), respectively. The FD-4 flux of HO+PD98059 group was decreased by 0.25 and 0.32 folds compared to only HO (*F* = 37.056, *P* < 0.0001) or PD98059 alone group (*F* = 45.087, *P* < 0.0001). The results demonstrated that the RACK1 inhibitor HO and ERK1/2 inhibitor PD98059 significantly reduced and abolished the increased intestinal permeability caused by *T*. *spiralis* infection and evidently improved intestinal epithelial integrity and barrier function. The results further confirmed that HO and PD98059 inhibited the binding of TsSPc to RACK1 and activation of p-ERK1/2 pathway, up-regulated and restored the expression of gut TJs proteins and gut epithelium integrity, consequently impeded larval invasion and reduced intestinal worm burdens.

In addition, the original full figures of SDS-PAGE and Western blot in this study were shown in S4 Fig.

## Discussion

Intestinal epithelial TJs as cell-cell adhesion are the semi-permeable barriers for ion, solute and water transport, also are the main barrier against foodborne parasite invasion and infection [17]. The TJs complex is mainly composed of claudins, occludin and Zos. The claudin family is composed of at least 24 proteins, among which differential expression characteristics determine tissue specific changes in epithelial resistance and cell bypass permeability [67]. Claudin-1 restricts ions from entering the epithelium and interacts with other claudins in the same and adjacent cells to form a barrier, avoiding lipid and protein diffusion [68], while claudin-2 exists in the leaky epithelium and allows cations to exit, which is consistent with the functional damage of TJs. Claudin-2 is highly expressed in sparse cell culture, with TJs leakage; while in the fusion monolayer, TJ is tight [69]. An *in vivo* study also showed that when the RNA of the truncated occludin construct was injected into *Xenopus* embryos, the truncated occludin was correctly targeted towards TJs and the barrier function was disrupted [70]. E-cad protein is involved in the development of adjacent junctions (AJs), starting from the initial cell-cell contact formed at the tips of filamentous and flaky foot protrusions. E-cad protein may also be used as a precursor to establish TJs at the top of AJs and gather around partner molecules, such as cell surface receptors, intracellular signaling molecules and oncoproteins [71].

Some studies showed that RACK1 scaffold protein had a specific function in the integrin-mediated activation of the MAPK/ERK cascade, and targeted the active ERK and focal adhesion. RACK1 was associated with the core kinases Raf, MEK and ERK of the ERK pathway, and the reduced expression of RACK1 lead to a decrease in ERK activity, which affected the adhesion function [72]. RACK1 promoted ERK1/2 phosphorylation and mediated multidrug resistance (MDR) cancer cell migration and invasion [73]. The down-regulation of RACK1 inhibited cell proliferation, invasion and migration of esophageal squamous cell carcinoma *in vitro* and *in vivo*, and the expression of RACK1 was negatively correlated with E-cad protein [74].

The MAPK/ERK signaling pathway is a well-known classical signaling pathway that regulates various cell activities, and it plays a crucial role in regulating intestinal epithelial barrier function [74,75]. Previous studies showed that foreign substances increased the expression of MAPK, leading to the disruption of the intestinal epithelium barrier [76]. The ESP of *T*. *spiralis* ML increased the phosphorylation of ERK and P38 in DC cells [77]. Recent studies showed that the serine protease in *T*. *spiralis* ML ESP reduced the expression of TJs protein claudin-1 through activating the p38 MAPK pathway in Caco-2 monolayer, thus destroyed the cell monolayer integrity [19].

In the present study, when intestinal sections of *T*. *spiralis*-infected mice at different times after infection were detected by IIFT with anti-rTsSPc serum and infection serum, immunostaining of intestinal epithelium was observed at 6 h, 12 h and 3 dpi, indicating that natural TsSPc was secreted and bound to intestinal epithelium at the early intestinal stage of *T*. *spiralis* infection. Previous studies showed that when the IIL were co-incubated with IECs monolayer, IIL penetrated into the monolayer and produced some proteases which entered into the IECs [42,78]. The results suggested that there was an interaction between TsSPc and gut epithelium cells, and TsSPc was involved in larval invasion of gut epithelium [8,9]. Therefore, in this study, we identified and characterized which kinds of IEC proteins bound to TsSPc by using Co-IP and mass spectrometry.

From metabolic process to structure, protein-protein interactions or protein complexes are indispensable in almost all cell processes. Co-IP, pull down assay and subsequent mass spectrometry (MS) are the standard methods for identifying protein-protein interactions, these techniques have been widely used to investigate the protein interaction between parasitic nematode and host [79,80]. In this study, the interaction between rTsSPc and Caco-2 cells was investigated by IIFT co-localization, GST pull-down, Co-IP and MS. The results of GST pull-down and MS showed the interaction of rTsSPc with RACK1 receptor in Caco-2 cells, which was further confirmed by Co-IP. Although MS results suggested that rTsSPc might directly interact with several proteins of Caco-2 cells, RACK1 had the highest coverage in MS-identified Caco-2 cell proteins. Other studies revealed that RACK1 regulated epithelial cell adhesion and tight junctions by activating the MAPK/ERK signaling pathway [72,74]. Therefore, we selected RACK1 for the following experiments. Our MS results also suggested that rTsSPc might interact with Plakophilin 1. Plakophilins have the significant functions for maintaining structural integrity of desmosomes, and are involved in protein synthesis, cell growth and proliferation [81], the possible interaction of rTsSPc and Plakophilin 1 is worthy of further research. In addition, the rTsSPc might bind with additional invisible protein bands of Caco-2 cells which were not identified in the GST pull-down test. The IIFT results showed that the co-localization of rTsSPc binding with RACK1 was localized at the cell–cell junctions, further indicating the interaction between rTsSPc and RACK1 in Caco-2 cells. The results suggested that rTsSPc interacting with RACK1 regulated further the expression of TJs and larval invasion of gut epithelium. The expression of RACK1 and MAPK/ERK1/2 related molecules in Caco-2 cells was ascertained by Western blot. The results showed that only active rTsSPc increased the expression level of RACK1 and p-EKR1/2, whereas MTsSPc and inactivated rTsSPc had no obvious effect on expression of RACK1 and p-EKR1/2 in Caco-2 cells. Previous studies revealed that serine proteases with enzymatic activity selectively cleaved and activated PAR2 receptor in intact cells, up-regulated the expression of PAR2, thereby activating the MAPK/ERK signaling pathway and down-regulating TJs (ZO-1, occludin, and claudin-1) to disrupt the epithelial barrier [82,83]. In this study, rTsSPc up-regulating the RACK1 expression and subsequently activating the MAPK/ERK pathway were also be related with its enzymatic activity. Compared to the PBS group, expression of RACK1 and p-EKR1/2 in Caco-2 cells pretreated with RACK1 inhibitor, anti-RACK1 antibody and MAPK/EKR1/2 pathway inhibitor had no evident changes after incubation with rTsSPc, suggesting that rTsSPc binding and interacting with RACK1 up-regulated the RACK1 expression, and consequently activated the downstream MAPK/EKR1/2 signaling pathway.

The IIFT, qPCR and Western blot were used to detect the expression changes of three TJs proteins and cadherin in epithelial cells after rTsSPc treatment. The results showed that transcription and expression levels of occludin, claudin-1 and E-cad in Caco-2 monolayer incubated with rTsSPc were significantly decreased, indicating that rTsSPc degraded the TJs proteins or down-regulated the expression of occludin, claudin-1 and E-cad. Moreover, rTsSPc also increased the paracellular permeability of Caco-2 cells, indicating that rTsSPc disrupted the integrity of epithelial cell monolayer [19]. However, the results of rTsSPc direct hydrolysis of Caco-2 cell TJs proteins revealed that the TJs proteins in Caco-2 cells was not hydrolyzed and degraded directly by rTsSPc, suggesting that rTsSPc disrupting gut epithelial integrity was not resulted from rTsSPc degrading TJs proteins, it was likely duo to rTsSPc reducing expression of TJs proteins through rTsSPc binding to RACK1 and activating ERK1/2 pathway in Caco-2 cells [84]. When the Caco-2 cells were pre-treated with RACK1 inhibitor HO, anti-RACK1 antibody, or ERK1/2 pathway inhibitor PD98059, and then incubated with rTsSPc, expression levels of occludin, claudin-1 and E-cad were restored, in other words, the two inhibitors and anti-RACK1 antibody suppressed and abrogated the rTsSPc down-regulating expression of occludin, claudin-1 and E-cad. The results further validated that rTsSPc reduced expression of TJs proteins in Caco-2 cells through rTsSPc binding to RACK1 receptor and activating the ERK1/2 pathway. The results of an *in vitro* larval invasion showed that RACK1 receptor inhibitor, anti-RACK1 antibody and ERK1/2 pathway inhibitors also obviously suppressed the IIL invasion of Caco-2 monolayer. Our results demonstrated that rTsSPc binding to RACK1 receptor in Caco-2 cells activated ERK1/2 signaling pathway, reduced the expression of TJs proteins and damaged the integrity and barrier function of gut epithelium, and facilitated the *T*. *spiralis* IIL invasion of gut epithelium. Therefore, TsSPc might be considered to be a candidate vaccine target against *T*. *spiralis* invasion and infection.

Animal challenge experiment showed that in *T*. *spiralis*-infected mice, the expression levels of RACK1 and ERK1/2 were clearly elevated, suggesting that *T*. *spiralis* stimulated RACK1 expression and activated ERK1/2 pathway. In infected PBS group, gut epithelial TJs proteins (occludin, and claudin-1) and adherens junction protein (E-cad) were significantly decreased; whereas expression levels of claudin-2, inflammatory cytokines (TNF-α, IL-1β, IL-4 and IL-10), and intestinal permeability were prominently increased. However, in infected mice pretreated by RACK1 receptor inhibitor HO and ERK1/2 pathway inhibitor PD98059, the expression levels of RACK1 and ERK1/2 was evidently suppressed and declined. The expression levels of gut TJs proteins, anti-inflammatory cytokines were significantly increased, while expression levels of claudin-2, pro-inflammatory cytokines were clearly declined again, and intestinal permeability also restored to near normal level of uninfected mice. The results demonstrated that HO and PD98059 suppressed and abrogated the changes of expression of RACK1 and ERK1/2, gut TJs proteins, inflammatory factors and epithelial permeability caused by *T*. *spiralis* infection, and restored gut epithelial barrier integrity. Moreover, HO and PD98059 significantly reduced intestinal IIL and adult burdens, further indicating that *T*. *spiralis* larval invasion of intestinal mucosa and development were related to the binding of TsSPc with RACK1 and activation of ERK1/2 pathway. Previous studies showed that RACK1 was involved in activation of ERK pathway, and the increase of RACK1 expression increased the ERK activity [72], which led the changes of expression levels gut TJs [85]. The animal experiment results further confirmed that TsSPc binding to RACK1 activated the ERK1/2 pathway, down-regulated the TJs expression, damaged integrity and barrier function of gut epithelia, consequently mediated the larval invasion at the early stage of *T*. *spiralis* infection.

*Trichinella spiralis* is a globally distributed foodborne parasitic nematode that affects both humans and animals. *T*. *spiralis* infection is caused by the ingestion of raw or undercooked infected animal meat. The IIL is the first invasive stage in *T*. *spiralis* life cycle [10]. Our results indicated that the disruption of enteral tight junctions caused by rTsSPc mediated the IIL invasion. When the larval invasion is hindered, the larval development will be interrupted, and *T*. *spiralis* infection be blocked [26,28]. Therefore, rTsSPc would be a potential candidate vaccine for preventing the *T*. *spiralis* invasion and infection. The anti-*Trichinella* vaccine provides a prospective strategy to control *Trichinella* infection in food animals and to ensure meat food safety [10].

However, this study still has some limitations. Although the results of this current study showed that rTsSPc did not directly hydrolyze the Caco-2 cell TJs proteins, other components of Caco-2 cell lysate might affect the rTsSPc activity (e.g., serpin). Thus, to confirm further the rTsSPc direct hydrolysis of Caco-2 cell TJs proteins, individual E-cad, occludin, claudin-1 and claudin-2 should be separately used for the rTsSPc direct hydrolysis assay in future study. The RACK1 receptor inhibitor harringtonolide (HO) was used to suppress the binding of rTsSPc with RACK1 in this study, but the knock-out and knock-down experiments of Caco-2 cell RACK1 receptor will confirm precisely the RACK1 specific role in *T*. *spiralis* invasion of gut epithelium. Moreover, the immune protection of vaccination of mice with rTsSPc against *T*. *spiralis* larval challenge is also necessary to be further evaluated.

In conclusion, rTsSPc especially binding and interacting with RACK1 in gut epithelium activated the downstream MAPK signaling pathway, reduced the expression of tight junctions proteins (occludin, claudin-1, and E-cad), increased the intestinal epithelial permeability and damaged the intestinal epithelial integrity and barrier function, consequently mediated *T*. *spiralis* larval invasion of host’s intestinal mucosa. *T*. *spiralis* infection also promoted the expression of pro-inflammatory cytokines (TNF-α and IL-1β) and decreased expression anti-inflammatory cytokines (IL-4 and IL-10), while HO (RACK1 receptor inhibitor) and PD98059 (ERK1/2 pathway inhibitor) abrogated the rTsSPc producing changes of expression level of RACK1 and ERK1/2, TJs proteins, inflammatory cytokines and permeability in Caco-2 cells and infected mouse intestine, impeded larval invasion of gut epithelium *in vitro* and *in vivo*. The results indicated that TsSPc participated in the *T*. *spiralis* invasion of host gut mucosa. The results are valuable to further understand *T*. *spiralis* invasion mechanism, and TsSPc may be considered as a candidate vaccine target against *T*. *spiralis* infection.

## Supporting information

S1 Table Primer sequences of gut epithelial tight junctions (TJs) and cytokine genes for qPCR. (DOCX)

S2 Table Protein mass spectrometry analysis of rTsSPc interacting Caco-2 cell proteins captured by GST pull-down. (DOCX)

S1 Fig Western blot identification of rTsSPc. **A**: SDS-PAGE analysis of rTsSPc. Lane M: protein marker; Lane 1: lysate of non-induced Origami proteins carrying pGEX-4T-1/TsSPc; Lane 2: lysate of induced Origami proteins carrying pGEX-4T-1/TsSPc; Lane 3: purified rTsSPc. **B**: Western blot identification of rTsSPc. Lane M: protein marker; Lane 1 and 4: Origami protein carrying pGEX-4T-1/TsSPc prior to induction was not probed by anti-GST tag antibody (lane 1) and anti-rTsSPc serum (lane 4); Lane 2 and 5: Origami protein carrying pGEX-4T-1/TsSPc after induction was recognized by anti-GST tag antibody (lane 2) and anti-rTsSPc serum (lane 5); Lane 3, 6 and 7: purified rTsSPc was recognized by anti-GST tag antibody (lane 3), anti-rTsSPc serum (lane 6) and *T*. *spiralis*-infected murine serum (lane 7). The arrow indicates rTsSPc with 51.18 kDa. Each experiment was carried out in triplicate.
(TIF)

S2 Fig CCK-8 assay of viability Caco-2 cells treated by rTsSPc. **A**: effect of various concentrations of rTsSPc on Caco-2 cell viability. **B**: viability of Caco-2 cells treated by 20 μg/ml of rTsSPc for various times. Each experiment was carried out in triplicate and the data were expressed as the mean ± SD of three independent experiments. * in the Figure represents *P* < 0.05 compared to the PBS control group.
(TIF)

S3 Fig Gene Ontology annotation and theoretical two-dimensional distribution of identified Caco-2 cells proteins. **A**: Gene ontology categories for identified human proteins. The identified proteins were classified into cellular component, molecular function and biological process by WEGO according to their GO signatures. The number of genes denotes the number of proteins with GO annotations. The left-hand shows the proportion in total genes with GO terms. **B**: Theoretical two-dimensional (MW, pI) distribution of identified Caco-2 cell proteins interacting with rTsSPc.
(TIF)

S4 Fig The original full figures of SDS-PAGE and Western blot in this study. (DOCX)
