# Supplementary material for: A novel Trichinella spiralis serine proteinase disrupted gut epithelial barrier and mediated larval invasion through binding to RACK1 and activating MAPK/ERK1/2 pathway
Source: PLoS Negl Trop Dis. 2024 Jan 8;18(1):e0011872. doi: 10.1371/journal.pntd.0011872 (PMC10798628; doi:10.1371/journal.pntd.0011872)
Supplement: S1 Table — (DOCX) [file pntd.0011872.s001.docx]

**Supporting information**

**S1 Table. Primer sequences of gut epithelial tight junctions (TJs) and cytokine**

**genes for qPCR**

| Gene names | Primer sequences (5′- 3′) | GenBank no. |
| --- | --- | --- |
| E-cad  (Human) | F: GCCTCCTGAAAAGAGAGTGGAAG | NM_004360.5 |
|  | R: TGGCAGTGTCTCTCCAAATCCG |  |
| Occludin  (Human) | F: ATGGCAAAGTGAATGACAAGCGG | XM_026274194.1 |
|  | R: CTGTAACGAGGCTGCCTGAAGT |  |
| Claudin-1  (Human) | F: GTCTTTGACTCCTTGCTGAATCTG | NM_021101.5 |
|  | R: CACCTCATCGTCTTCCAAGCAC |  |
| Claudin-2  (Human) | F: GTGACAGCAGTTGGCTTCTCCA | NM_001171095.2 |
|  | R: GGAGATTGCACTGGATGTCACC |  |
| GAPDH  (Human) | F: GTCTCCTCTGACTTCAACAGCG | NM_002046.7 |
|  | R: ACCACCCTGTTGCTGTAGCCAA |  |
| E-cad  (Mouse) | F: GGTCATCAGTGTGCTCACCTCT | NM_009864.3 |
|  | R: GCTGTTGTGCTCAAGCCTTCAC |  |
| Occludin  (Mouse) | F: TGGCAAGCGATCATACCCAGAG | NM_001360536.1 |
|  | R: CTGCCTGAAGTCATCCACACTC |  |
| Claudin-1  (Mouse) | F: GGACTGTGGATGTCCTGCGTTT | NM_016674.4 |
|  | R: GCCAATTACCATCAAGGCTCGG |  |
| Claudin-2  (Mouse) | F: AGGACTTCCTGCTGACATCCAG | NM_001410421.1 |
|  | R: AATCCTGGCAGAACACGGTGCA |  |
| GAPDH  (Mouse) | F: CATCACTGCCACCCAGAAGACTG | NM_001411840.1 |
|  | R: ATGCCAGTGAGCTTCCCGTTCAG |  |
| TNF-α  (Mouse) | F: CCCTCACACTCAGATCATCTTCT | NM_013693.3 |
|  | R: GCTACGACGTGGGCTACAG |  |
| IL-1β  (Mouse) | F: AGCTCTCCACCTCAATGGAC | NM_008361.4 |
|  | R: ATCATTGCGTGGGATCTTGA |  |
| IL-4  (Mouse) | F: TTGTCATCCTGCTCTTCTTTCT | NM_021283.2 |
|  | R: CTGTGGTGTTCTTCGTTGCT |  |
| IL-10  (Mouse) | F: CCCTTTGCTATGGTGTCCTT | Nm_010548.2 |
|  | R: TGGTTTCTCTTCCCAAGACC |  |
