# Supplementary material for: A novel Trichinella spiralis serine proteinase disrupted gut epithelial barrier and mediated larval invasion through binding to RACK1 and activating MAPK/ERK1/2 pathway
Source: PLoS Negl Trop Dis. 2024 Jan 8;18(1):e0011872. doi: 10.1371/journal.pntd.0011872 (PMC10798628; doi:10.1371/journal.pntd.0011872)
Supplement: S2 Table — (DOCX) [file pntd.0011872.s002.docx]

**S2 Table. Protein mass spectrometry analysis of rTsSPc interacting Caco-2 cell proteins captured by GST pull-down**

| **Protein name** | **Protein IDs** | **pI** | **Gene name** | **Theor. MW**  **(kDa)** | **peptides** | **Coverage**  **(%)** | **Go annotations** |
| --- | --- | --- | --- | --- | --- | --- | --- |
| Protein POF1B | Q8WVV4 | 6.32 | POF1B | 68.0 | 1 | 1.70 | Bicellular tight junction assembly; epithelial cell morphogenesis; actin filament organization; actin cytoskeleton organization; actin filament binding; desmosome; adherens junction; bicellular tight junction; actin filament. |
| Desmocollin 1 | Q9HB00 | 5.53 | DSC1 | 93.8 | 1 | 1.79 | Homophilic cell adhesion via plasma membrane adhesion molecules; calcium ion binding; plasma membrane; integral component of membrane. |
| UPF0764 protein C16orf89 | Q6UX73 | 6.19 | 146556 | 45.4 | 1 | 1.74 | Protein homodimerization activity; membrane; cytosol; extracellular exosome. |
| Plakophilin 1 | A0A024R952 | 8.97 | PKP1 | 80.4 | 1 | 2.07 | Cell-cell adhesion; plasma membrane; nucleoplasm; desmosome. |
| Chloride intracellular channel protein | A0A1U9X8Y4 | 5.26 | **-** | 26.8 | 2 | 8.33 | Regulation of ion transmembrane transport; voltage-gated ion channel activity; chloride channel activity; plasma membrane; cytoplasm; nuclear membrane; chloride channel complex. |
| Prohibitin | J3KPX7 | 9.83 | PHB2 | 33.2 | 2 | 5.7 | Plasma membrane; mitochondrial inner membrane; mitochondrion. |
| Receptor of activated protein C kinase 1 | P63244 | 7.69 | RACK1 | 35.1 | 7 | 22.4 | Negative regulation of peptidyl-serine phosphorylation; cellular response to growth factor stimulus; positive regulation of gastrulation. |

**The black font in the table represents the proteins screened from the protein band of 36 kDa by LC-MS/MS, while the blue font represents the proteins screened from the protein band of 55 kDa.**
