# Supplementary figures and images for: A novel Trichinella spiralis serine proteinase disrupted gut epithelial barrier and mediated larval invasion through binding to RACK1 and activating MAPK/ERK1/2 pathway
Source: PLoS Negl Trop Dis. 2024 Jan 8;18(1):e0011872. doi: 10.1371/journal.pntd.0011872 (PMC10798628; doi:10.1371/journal.pntd.0011872)

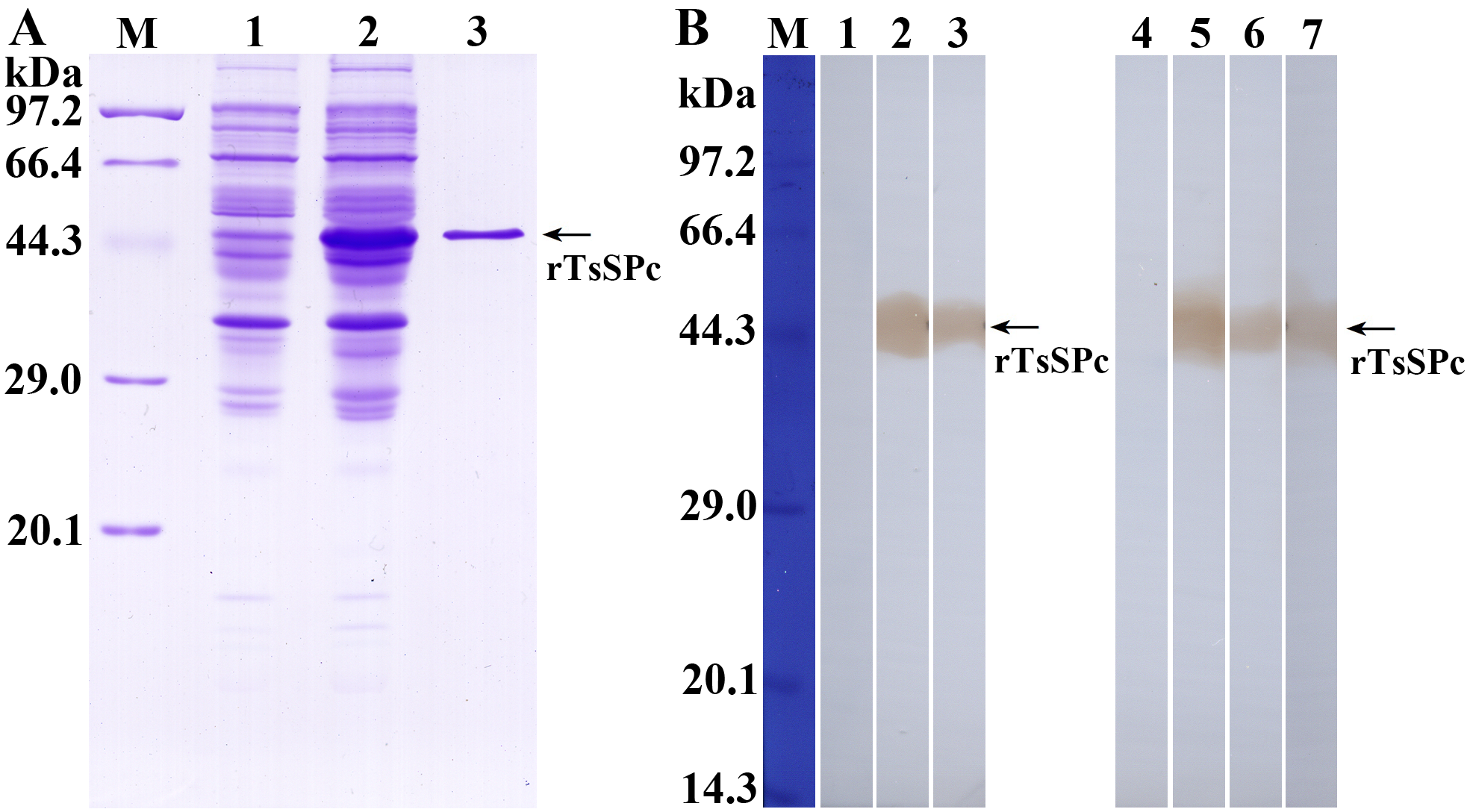

Supplement: S1 Fig — A: SDS-PAGE analysis of rTsSPc. Lane M: protein marker; Lane 1: lysate of non-induced Origami proteins carrying pGEX-4T-1/TsSPc; Lane 2: lysate of induced Origami proteins carrying pGEX-4T-1/TsSPc; Lane 3: purified rTsSPc. B: Western blot identification of rTsSPc. Lane M: protein marker; Lane 1 and 4: Origami protein carrying pGEX-4T-1/TsSPc prior to induction was not probed by anti-GST tag antibody (lane 1) and anti-rTsSPc serum (lane 4); Lane 2 and 5: Origami protein carrying pGEX-4T-1/TsSPc after induction was recognized by anti-GST tag antibody (lane 2) and anti-rTsSPc serum (lane 5); Lane 3, 6 and 7: purified rTsSPc was recognized by anti-GST tag antibody (lane 3), anti-rTsSPc serum (lane 6) and T. spiralis-infected murine serum (lane 7). The arrow indicates rTsSPc with 51.18 kDa. Each experiment was carried out in triplicate. (TIF) [file pntd.0011872.s003.tif]

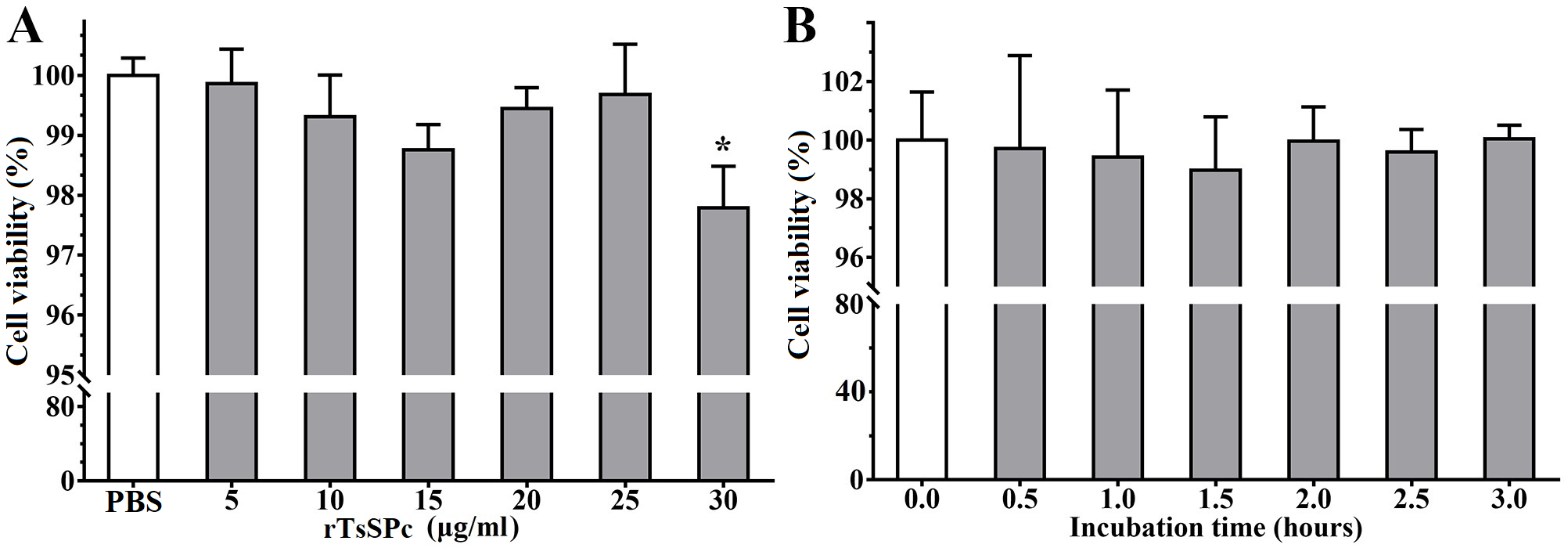

Supplement: S2 Fig — A: effect of various concentrations of rTsSPc on Caco-2 cell viability. B: viability of Caco-2 cells treated by 20 μg/ml of rTsSPc for various times. Each experiment was carried out in triplicate and the data were expressed as the mean ± SD of three independent experiments. * in the Figure represents P < 0.05 compared to the PBS control group. (TIF) [file pntd.0011872.s004.tif]

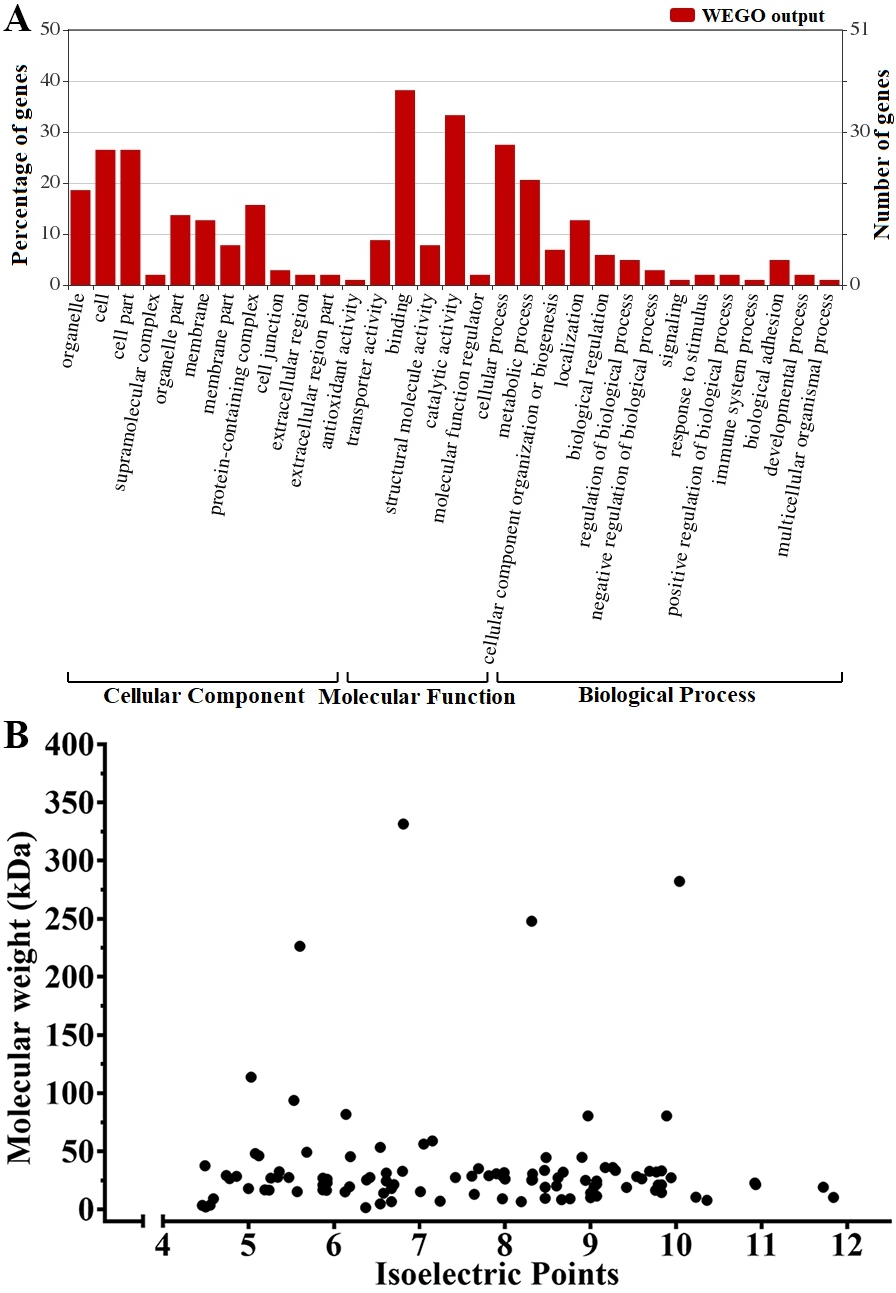

Supplement: S3 Fig — A: Gene ontology categories for identified human proteins. The identified proteins were classified into cellular component, molecular function and biological process by WEGO according to their GO signatures. The number of genes denotes the number of proteins with GO annotations. The left-hand shows the proportion in total genes with GO terms. B: Theoretical two-dimensional (MW, pI) distribution of identified Caco-2 cell proteins interacting with rTsSPc. (TIF) [file pntd.0011872.s005.tif]
