# Supplementary material for: A novel Trichinella spiralis serine proteinase disrupted gut epithelial barrier and mediated larval invasion through binding to RACK1 and activating MAPK/ERK1/2 pathway
Source: PLoS Negl Trop Dis. 2024 Jan 8;18(1):e0011872. doi: 10.1371/journal.pntd.0011872 (PMC10798628; doi:10.1371/journal.pntd.0011872)
Supplement: S4 Fig — (DOCX) [file pntd.0011872.s006.docx]

**S4 Fig. Original image of full gel/blot in PNTD-D-23-01129 R1**


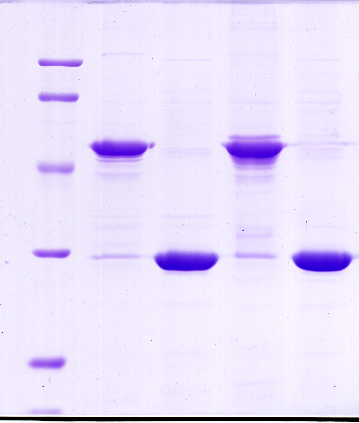


SDS-PAGE image of Fig 2


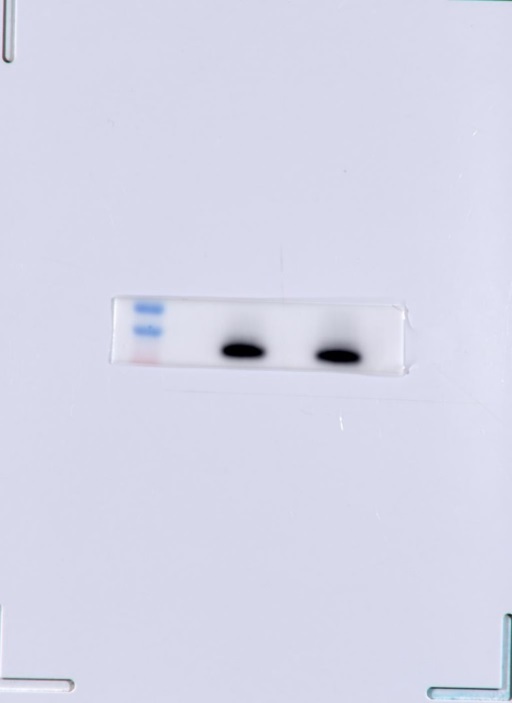


Figure 3B. Original image of RACK1


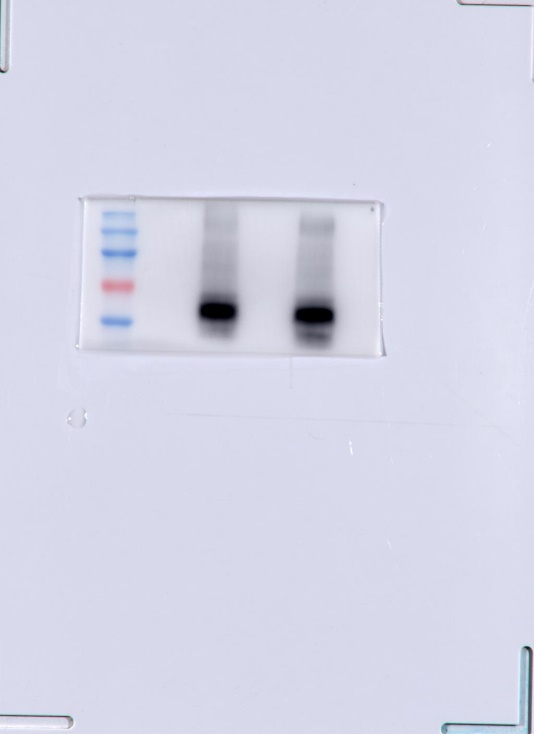


Figure 3B. Original image of TsSPc





Fig 4D (E-cad)





Fig 4D (occludin)





Fig 4D (claudin-1)





Fig 4D (claudin-2)





Fig 4D (GAPDH)





Fig 5A (RACK1)





Fig 5A (GAPDH)





Fig 5B (p-ERK1/2)





Fig 5B (ERK1/2)





Fig 5B (GAPDH)





Fig 6A (E-cad)





Fig 6A (occludin)





Fig 6A (claudin-1)





Fig 6A (claudin-2)





Fig 6A (GAPDH)





Fig 8D (E-cad)





Fig 8D (occludin)





Fig 8D (claudin-1)





Fig 8D (claudin-2)





Fig 8D (GAPDH)





Fig 9A (RACK1)





Fig 9A (GAPDH)





Fig 9B (p-ERK1/2)





Fig 9B (ERK1/2)





Fig 9B (GAPDH)





Fig 11A (RACK1)


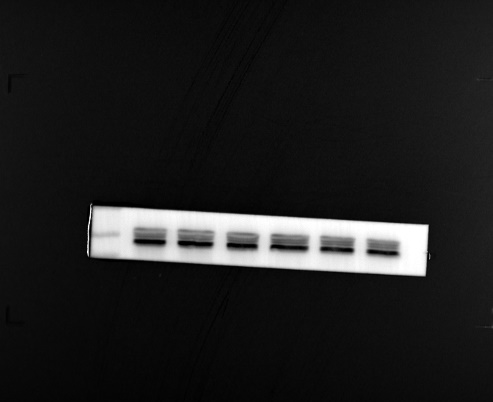


Fig 11A (p-ERK1/2)


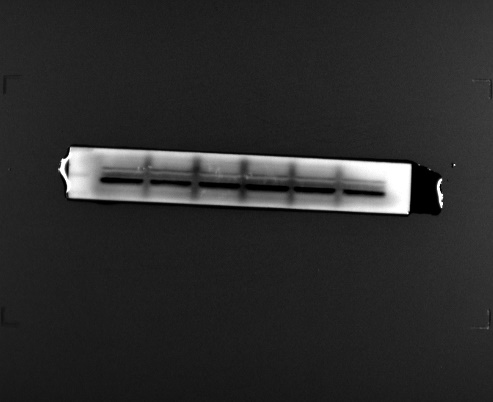


Fig 11A (ERK1/2)


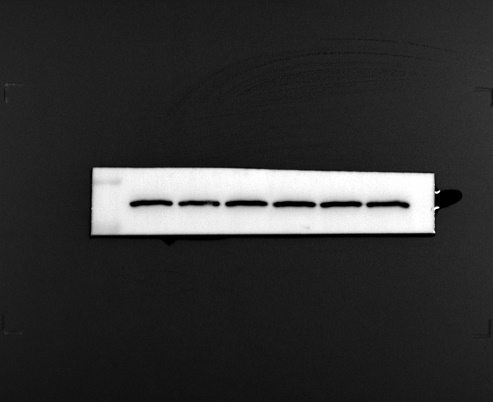


Fig 11A (GAPDH)


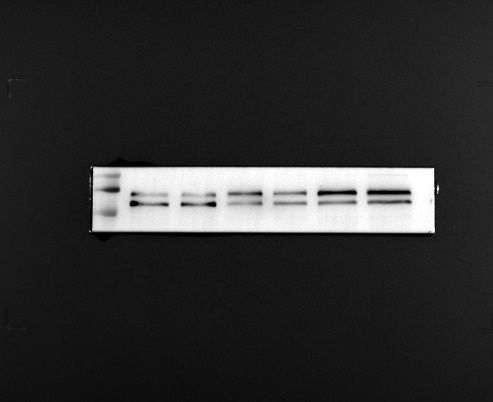


Fig 12B (E-cad)


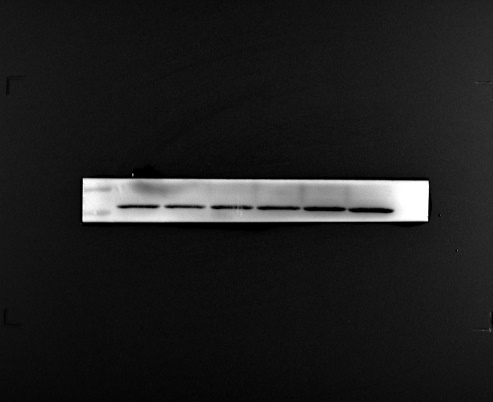


Fig 12B (occludin)





Fig 12B (claudin-1)





Fig 12B (claudin-2)


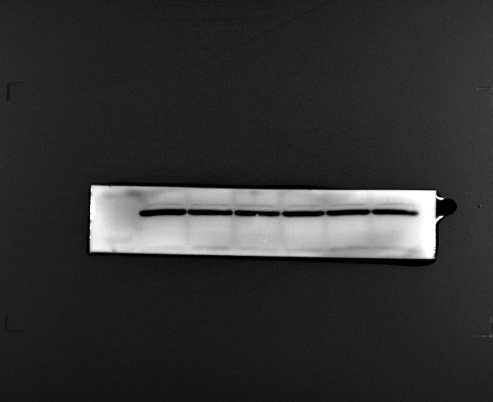


Fig 12B (GAPDH)


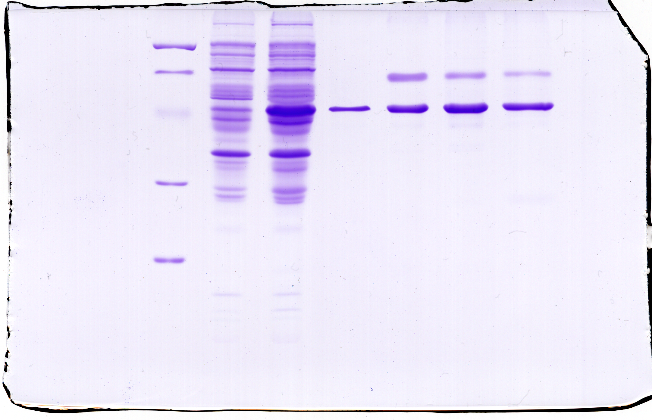


SDS-PAGE image of S1 Fig A


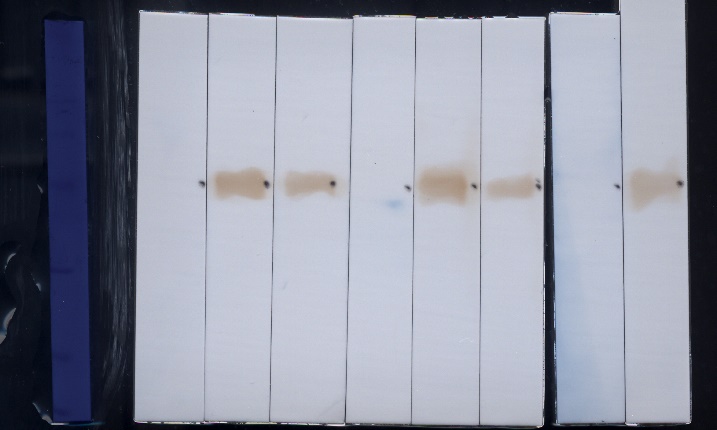


Western blot image of S1 Fig B
